# Engineering and design of promising T-cell-based multi-epitope vaccine candidates against leishmaniasis

**DOI:** 10.1038/s41598-023-46408-1

**Published:** 2023-11-08

**Authors:** Esmaeil Roohparvar Basmenj, Mahshid Arastonejad, Mina Mamizadeh, Mahsa Alem, Mahdi KhalatbariLimaki, Shadan Ghiabi, Ali Khamesipour, Hamidreza Majidiani, Morteza Shams, Hamid Irannejad

**Affiliations:** 1 Biophysics Department, Faculty of Biological Sciences, Tarbiat Modares University, Tehran, Iran; 2 Department of Human and Molecular Genetics, Virginia Commonwealth University, Richmond, VA USA; 3 Department of Dermatology, School of Medicine, Ilam University of Medical Sciences, Ilam, Iran; 4 Zoonotic Diseases Research Center, Ilam University of Medical Sciences, Ilam, Iran; 5 Department of Microbiology, Faculty of Veterinary Medicine, Urmia University, Urmia, Iran; 6 Department of Pharmaceutical Sciences, School of Pharmacy, Guilan University of Medical Sciences, Rasht, Iran; 7 Faculty of Veterinary Medicine, Science and Research Branch, Islamic Azad University, Tehran, Iran; 8 Center for Research and Training in Skin Diseases and Leprosy, Tehran University of Medical Sciences, Tehran, 14155-6383 Iran; 9 Healthy Aging Research Centre, Neyshabur University of Medical Sciences, Neyshabur, Iran; 10 Department of Basic Medical Sciences, Neyshabur University of Medical Sciences, Neyshabur, Iran; 11 Department of Medicinal Chemistry, Faculty of Pharmacy, Mazandaran University of Medical Sciences, Sari, Iran; 12 Pharmaceutical Sciences Research Center, Mazandaran University of Medical Sciences, Sari, Iran

**Keywords:** Protein design, Protein structure predictions, Parasite host response

## Abstract

Cutaneous leishmaniasis (CL) is a very common parasitic infection in subtropical areas worldwide. Throughout decades, there have been challenges in vaccine design and vaccination against CL. The present study introduced novel T-cell-based vaccine candidates containing IFN-γ Inducing epitopic fragments from *Leishmania major* (*L. major*) glycoprotein 46 (gp46), cathepsin L-like and B-like proteases, histone H2A, glucose-regulated protein 78 (grp78) and stress-inducible protein 1 (STI-1). For this aim, top-ranked human leukocyte antigen (HLA)-specific, IFN-γ Inducing, antigenic, CD_4_^+^ and CD_8_^+^ binders were highlighted. Four vaccine candidates were generated using different spacers (AAY, GPGPG, GDGDG) and adjuvants (RS-09 peptide, human IFN-γ, a combination of both, *Mycobacterium tuberculosis* Resuscitation promoting factor E (RpfE)). Based on the immune simulation profile, those with RS-09 peptide (Leish-*App*) and RpfE (Leish-*Rpf*) elicited robust immune responses and their tertiary structure were further refined. Also, molecular docking of the selected vaccine models with the human toll-like receptor 4 showed proper interactions, particularly for Leish-*App*, for which molecular dynamics simulations showed a stable connection with TLR-4. Upon codon optimization, both models were finally ligated into the pET28a( +) vector. In conclusion, two potent multi-epitope vaccine candidates were designed against CL and evaluated using comprehensive in silico methods, while further wet experiments are, also, recommended.

## Introduction

**Figure 1:**
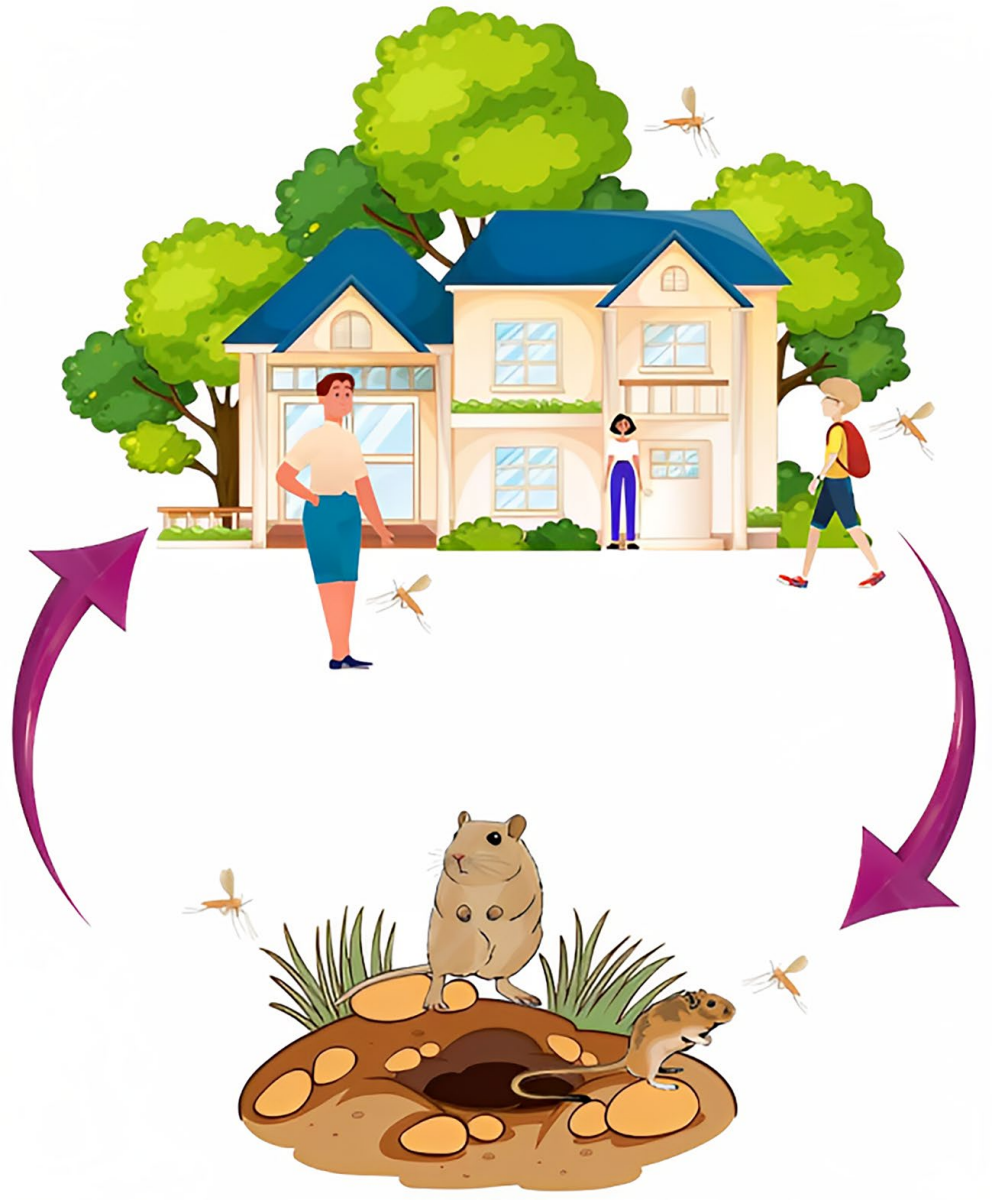
Zoonotic transmission cycle of *L. major* by *Phlebotomus* sandflies, involving gerbils and humans.

Leishmaniases are a heterogeneous group of parasitic diseases with considerable mortality, just second to the malaria, caused by the arthropod-borne flagellated protozoan, *Leishmania* spp., being vectored by the bites of infected phlebotomine sand flies^1,2^. *Leishmania* species are obligatory intracellular organisms, with macrophages as the main target cell^3^. Leishmaniases are represented with diverse clinical manifestations ranging from cutaneous lesions to the mucocutaneous involvement and fatal systemic infection. In total, 350 million individuals in 98 countries are threatened by different types of leishmaniasis. Cutaneous leishmaniasis (CL) is the most common form of the Disease, affecting 0.7–1.2 million people annually; Several species are known as the causative agents of CL, including *Leishmania* (L.) *major*, *L. tropica*, *L. aethiopica*, *L. infantum* and *L. donovani* with specific distribution in the Middle East (Iran, Iraq, Syria and Afghanistan), North Africa (Algeria) and South America (Brazil and Colombia)^4^. Rodents and humans are the principal reservoir hosts for *L. major* and *L. tropica* infections (Fig. 1). Actually, CL is a public health concern in some of the endemic areas and neither reservoir (humans, rodents) nor vector control is effective, Moreover, the available treatments are not safe and the efficacy is not high^5^. This issue justifies an urgent need for the discovery of novel vaccine targets and subsequent development of unprecedented vaccine candidates.

A variety of antigenic proteins are expressed in each life stages of *Leishmania*, i.e. amastigotes (within macrophages and dendritic cells), promastigotes and some intermediate forms (within sand fly gut), which might be directed towards vaccination strategies against leishmaniasis^6^. In previous studies, multiple *L. major* antigens have been discovered and used in immunization studies against leishmaniasis, most of which are conserved among other *Leishmania* species. Among these, some have been recognized with promising preventive effects, comprising glycoprotein 46 (gp46), cathepsin L-like and B-like proteases, histone H2A, glucose-regulated protein 78 (grp78) and stress-inducible protein 1 (STI-1)^7,8^. Cell-mediated immune responses may act as a double-edged sword during the course of CL lesion development and subsequent healing process; the IFN-γ cytokine, induced by type-I helper T-cells (Th1), is a potent killer of *Leishmania* parasites within the macrophages, through activation of macrophages and subsequent upsurge in nitric oxide (NO) and reactive oxygen species (ROS), while on the other hand Th2-mediated cytokines (Interleukin-4 (IL-4), IL-5 and IL-10) favor parasite survival, devastating the course of the infection^9–11^. Thus, in-depth exploration of antigenic vaccine candidates is advantageous to find novel immunodominant peptide targets, so-called as immunogenic epitopes.

Conventional vaccinology requires a vast amount of specialized biomolecular equipment, trained laboratory personnel, appropriate animal models, followed by long-lasting experimental intervals^12–14^. Advances in the biomedical informatics associated with novel genome sequencing technologies have led to the progression in biological databases and computational methods. This ongoing flow of information will increase our knowledge of host–pathogen interactions and aid in vaccinology-related research against infectious and non-infectious diseases. Such modalities give us the chance to recognize and organize novel antigenic proteins and specific B- and T-cell epitopes towards the development of safer and more effective next-generation vaccine candidates in a cost- and time-effective manner^15–18^. Therefore, in silico exploration of vaccine candidate antigens and their immunodominant epitope regions can provide important insights for future vaccinology studies. The present computer-based investigation was aimed at identification of immunogenic, IFN-γ-inducing epitopes with specific binding to the human leukocyte antigen (HLA) alleles in six antigenic proteins of *L. major*, *i.e.* gp46, CatL, CatB, H2A, grp78 and STI-1, in order to assemble novel T-cell-based multi-epitope vaccine candidates (MEVCs) against leishmaniasis.

## Results

### Selected human MHC-binders and engineering of MEVCs

**Figure 2:**
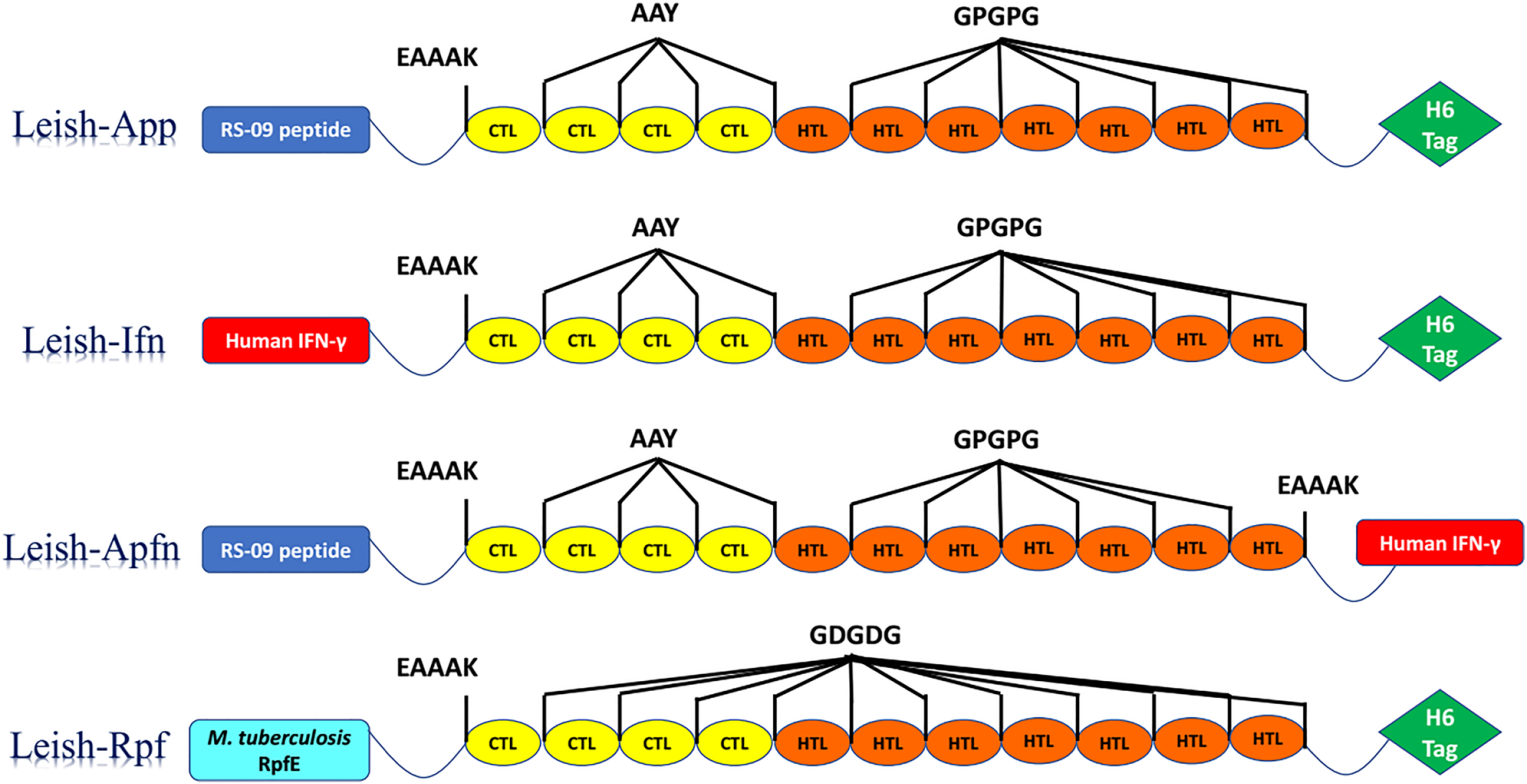
Engineering and arrangement of CTL and HTL epitopes along with used linkers, adjuvants and His-tag sequences in each designed MEVC.

Following strict prediction and screening procedures applied to the six *L. major* vaccine candidate antigens, four CTL epitopes (CatB_2-10_, gp46_3-11_, gp46_27-35_, gp46_13-21_,) and seven HTL epitopes (CatB_12-26_, grp78_38-52_, grp78_37-51_, gp46_136-150_, gp46_137-151_, gp46_133-147_, STI-1_10-24_) were finally highlighted for designing MEVCs. In the present in silico study, four potent MEVCs were designed, based on different adjuvants and linkers, as follow: **Seq1 or Leish-*App***) a TLR-4 agonist as RS-09 synthetic peptide (APPHALS) (N-terminal), His-Tag sequence (C-terminal), in addition to AAY and GPGPG linkers; **Seq2 or Leish-*Ifn***) human IFN-*γ* as a genetic adjuvant (N-terminal), His-Tag sequence (C-terminal) with AAY and GPGPG linkers; **Seq3 or Leish-*Apfn***) APPHALS (N-terminal) and human IFN-*γ* as adjuvants, with AAY and GPGPG linkers, and **Seq4 or Leish-*Rpf***) *M. tuberculosis* resuscitation-promoting factor E (RpfE) as adjuvant (N-terminal), His-Tag sequence (C-terminal), and GDGDG as linker. In Seq1-3 constructs, “AAY” and “GPGPG” linkers were used to adjoin CTL and HTL epitopes, respectively, whereas in Seq4, “GDGDG” linker was used for both kind of epitopes. Also, “EAAAK” linker was used to connect sequences belonging to adjuvants to the rest of the MEVCs. The full details of the designed MEVC constructs are illustrated in Fig. 2.

### Prediction of antigenicity, allergenicity, solubility and physico-chemical properties

Among four different MEVCs designed in the present study, the highest antigenicity predicted by VaxiJen server belonged to “Leish-*App*” and “Leish-*Rpf*” vaccine sequences, with 0.8376 and 0.7866, respectively. All MEVCs were approved to be non-allergenic in nature, as substantiated by AllergenFP v1.0 and AllerTOP v2.0 servers. The above-mentioned vaccine candidates possessed the highest solubility scores, with 0.545 (Leish-*App*) and 0.626 (Leish-*Rpf*), whilst the solubility of Leish-*Apfn* and Leish-*Ifn* vaccine candidates was 0.313 and 0.282, respectively, which was lower than the threshold (0.45). The lengths of Seq1-4 candidates, predicted using ProtParam server, were 218, 377, 366 and 391 residues, respectively. Also, Leish-*Ifn* and Leish-*App* had the highest and lowest MW, with 40.25 and 21.59 kDa, respectively. All of the designed candidates had extreme thermotolerance, as demonstrated by high aliphatic index. Moreover, all candidates, except Leish-*App*, had negative GRAVY scores, hence were recognized as hydrophilic in nature. Since the instability index of all vaccine candidates in the present study was below 40, they were assigned as “stable” polypeptides. Details of the physico-chemical properties of four designed MEVCs are provided in Table 1.

**Table 1:** Comparable physico-chemical characteristics of four designed MEVCs against CL, predicted using ExPASy ProtParam web server.

MEVCs	No. of residues	MW*	Theoretical pI	+ charged residues	− charged residues	Half-life**	Instability index	Aliphatic index	GRAVY***
Leish-*App*	218	21594.66	4.76	6	13	4.4 h	17.34 (stable)	87.94	0.401
Leish-*Ifn*	377	40251.18	8.15	35	32	30 h	22.18 (stable)	82.39	− 0.023
Leish-*Apfn*	366	39372.05	8.51	36	33	4.4 h	21.42 (stable)	85.66	− 0.102
Leish-*Rpf*	391	38958.78	3.93	16	51	30 h	29.54 (stable)	76.04	− 0.061

*MW: molecular weight (Dalton).

**It means estimated half-life in mammalian reticulocytes, in vitro.

***Grand Average of Hydropathicity.

### Structural analyses of different MEVCs

Regarding secondary structure analysis outputs using GOR IV online tool, random coils were the most prominent structures found in all MEVCs, with the exception of Leish-*Apfn*, where alpha helices were abundant (47.54%). The highest percentage of random coils were predicted in Leish-*Rpf* (59.34%), Leish-*App* (50.92%) and Leish-*Ifn* (44.83%), respectively (Supplementary Figures). In the following, a homology modelling prediction by I-TASSER server was used to illustrate the 3D model of the engineered MEVCs. A highest C-score usually supports a more reliable predicted model. On this basis, Leish-*App* model 1 (C-score:  − 2.62), Leish-*Ifn* model 4 (C-score:  − 2.28), Leish-*Apfn* model 2 (C-score:  − 1.09) and Leish-*Rpf* model 3 (C-score:  − 2.62) were selected among top five predicted models by I-TASSER web server (Supplementary Figures).

### Simulation of the immune responses using MEVCs

**Figure 3:**
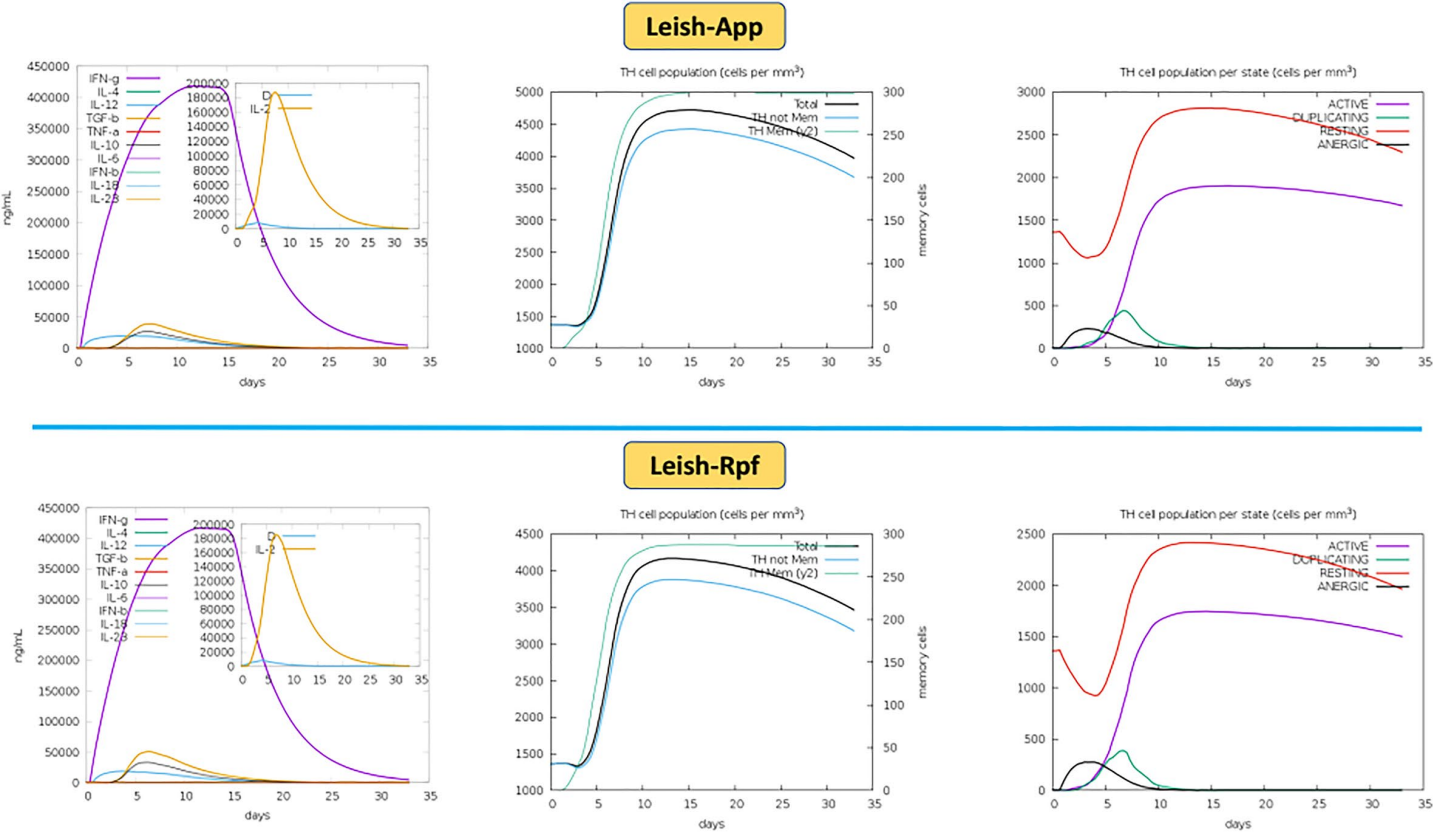
The elicited cytokines and Th-cell immune profile of administered Leish-*App* and Leish-*Rpf* vaccine candidates.

Using C-ImmSim web server, three major immune-related parameters, including cytokine induction, Th cell population (cells per mm^3^) and Th cell population per state (cells per mm^3^) were evaluated for each vaccine candidate. Based on cytokines, IFN-γ in all candidates ranged between 4,00,000 ng/ml to 4,50,000 ng/ml, while IL-2, another Th1-specific cytokine, was higher (over 1,80,000 ng/ml) in Leish-*App* and Leish-*Rpf* vaccine candidates and lower rates (between 1,40,000 and 1,60,000 ng/ml) were elicited by the two remaining constructs. With respect to the Th cell population type, the highest number of specific memory T cells belonged to Leish-*App* (300 per mm^3^) and Leish-*Rpf* (between 250 and 300 per mm^3^) vaccine candidates. Additionally, Th cell immune responses by Leish-*App* and Leish-*Rpf* vaccine candidates showed the highest numbers of duplicating (near 500) and active (over 1500) cells per mm^3^. As compared with gp46 and LeIF as potent *Leishmania* vaccine candidate antigens, similar cytokine profile was elicited by the selected MEVCs, showing their potency (Supplementary Figures). Thus, based on the immune simulation results, Leish-*App* and Leish-*Rpf* vaccine models were chosen as the best immunogenic MEVCs for further analyses (Fig. 3).

### Tertiary model refinement and validation

**Figure 4:**
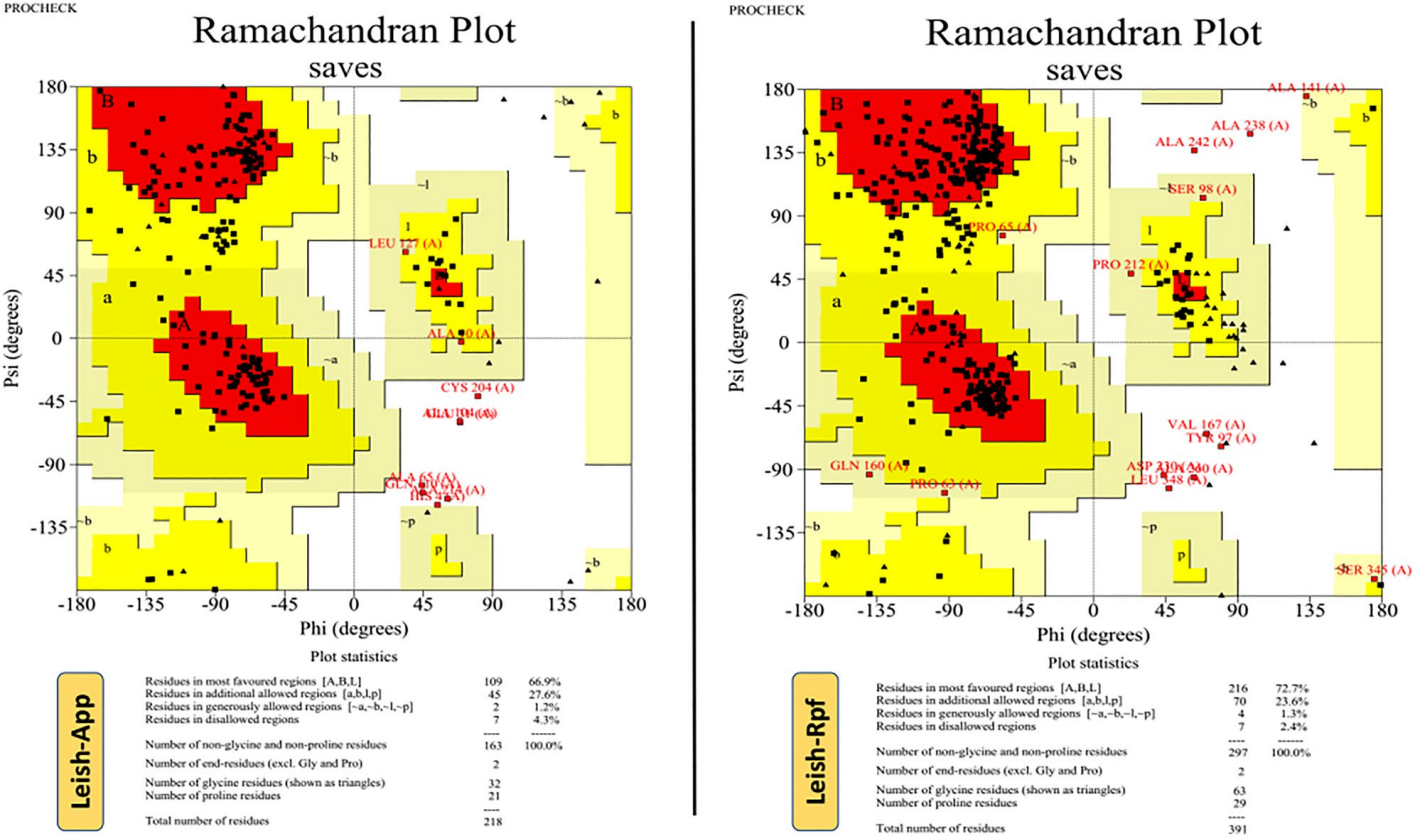
The validation of the 3D vaccine models upon refinement using Ramachandran plot analysis by PROCHECK web tool.

Pertinent to the GalaxyRefine server outputs, Leish-*App* model 4 was selected as the best model with acceptable rehashing and structural relaxation parameters, among other four refined models. The evaluated parameters for this model were as follows: GDT-HA of 0.9472, RMSD of 0.442, MolProbity of 2.513, Clash score of 24.8, Poor rotamers of 0.0 and Rama favored of 86.6. Regarding Leish-*Rpf* vaccine candidate, the refined model number 3 was selected, having GDT-HA of 0.9418, RMSD of 0.440, MolProbity of 2.380, Clash score of 17.3, Poor rotamers of 0.0 and Rama favored of 85.9. The quality improvement of the refined models for both candidates, in comparison with the crude models, was confirmed by the PROCHECK online tool. In the case of Leish-*Rpf*, the percentage of residue allocation in the crude and refined models, were as follows: 49.8% vs 72.7% (in the most favored regions), 42.1% vs 23.6% (in additional allowed regions), 5.7% vs 1.3% (in generously allowed regions) and 2.4% vs 2.4% (in disallowed regions). The crude model of Leish-*App* showed 72 residues (44.2%) in the most favored regions, followed by 76 residues (46.6%) in additional allowed regions, 7 residues (4.3%) in generously allowed regions as well as 8 residues (4.9%) in disallowed regions, whereas they were improved as 109 (66.9%), 45 (27.6%), 2 (1.2%) and 7 (4.3%) in the refined Leish-*App* model (Fig. 4).

### Molecular docking analysis using human TLR-4 as receptor

**Figure 5:**
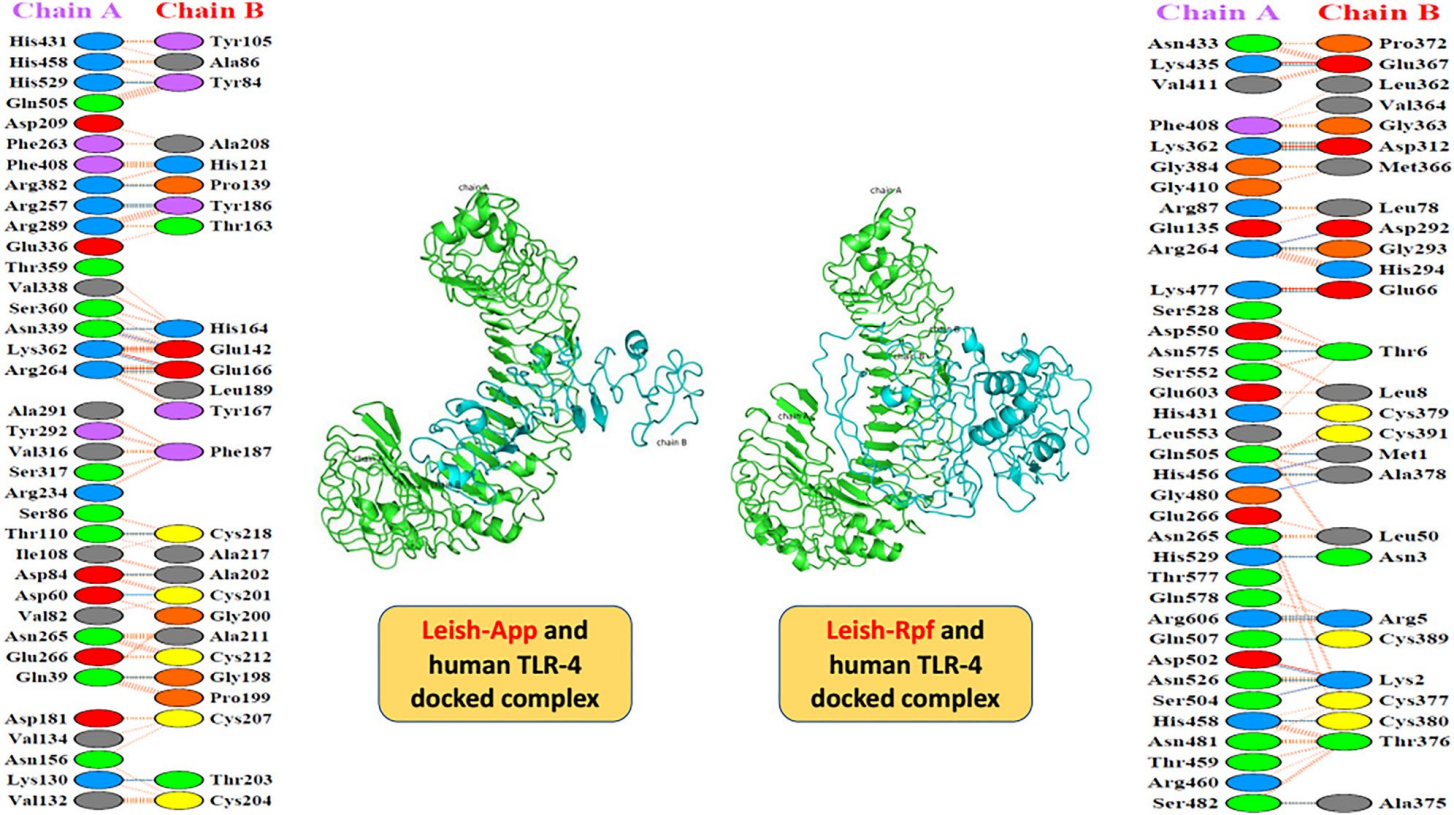
The representation of the whole docked complexes (vaccine-TLR4) and their respective residue-by-residue interactions. Regarding Leish-*App*/TLR-4 complex, there were 217 members with the lowest energy of  − 1121.4, while in Leish-*Rpf*/TLR-4 complex, there observed 40 members with the lowest energy of  − 863.1.

Based on the ClusPro 2.0 web server for the protein–protein docking, the most populated docking clusters for Leish-*App*/TLR4 and Leish-*Rpf*/TLR4 had 217 and 40 members, respectively, with the highest binding scores of  − 1121.4 and  − 863.1. The non-bonded contacts (*e.g.*, Van der Waals forces, hydrophobic forces, etc.) constituted the most part of interactions in Leish-*App*/TLR4 and Leish-*Rpf*/TLR4, with 154 and 189 contacts, respectively. The vaccines-receptor interactions have been illustrated in details in Fig. 5.

### Stability of vaccine construct complexed with TLR-4 receptor

**Figure 6:**
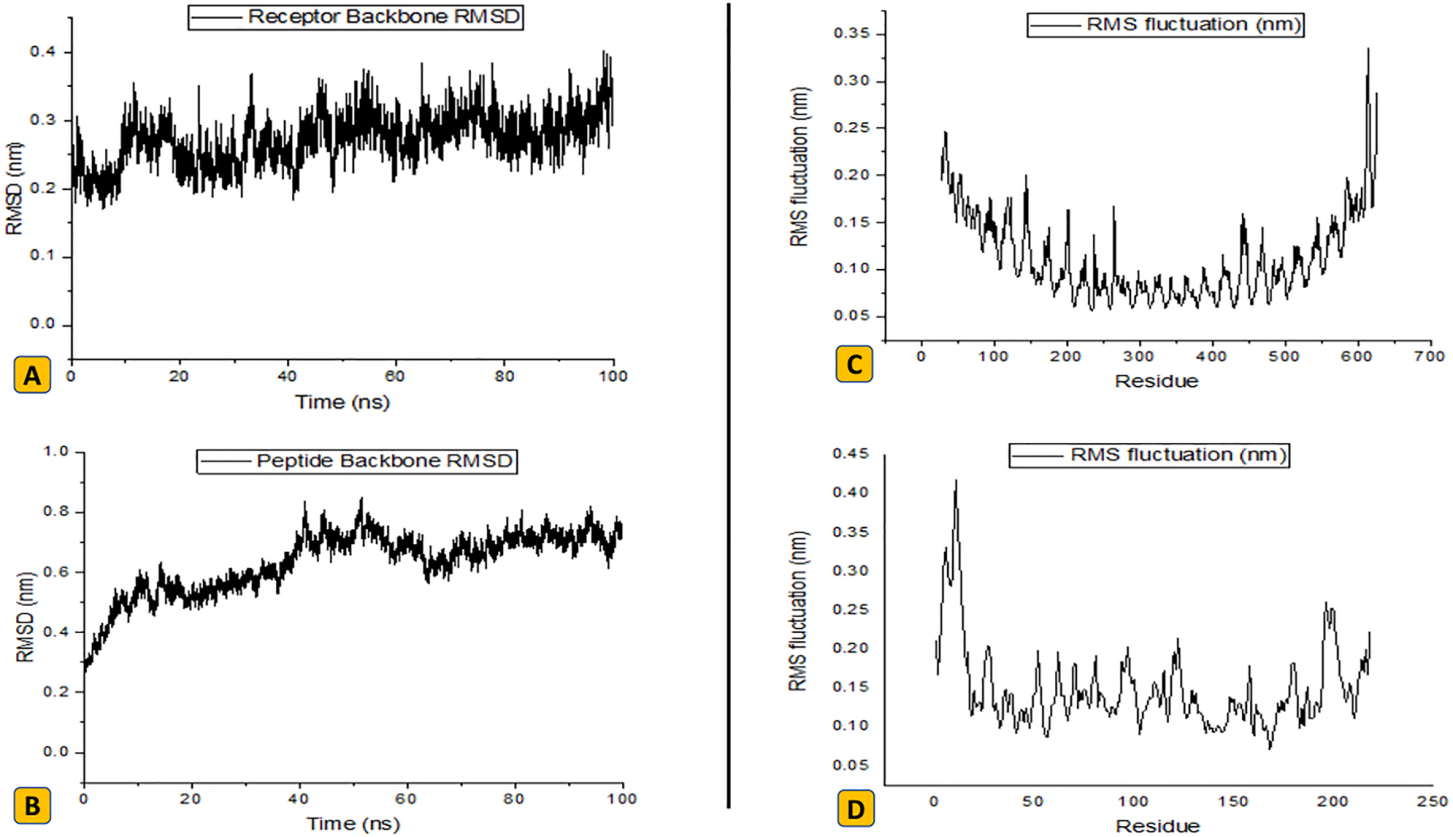
The MD simulation study for *Leish*-APP/TLR-4 complex. (A) RMSD plot analysis for TLR-4 receptor protein. (B) RMSD plot analysis for *Leish*-App peptide. (C) RMSF plot residue analysis for TLR-4 receptor protein. (D) RMSF plot residue analysis for *Leish*-App peptide.

The simulation of the *Leish*-App-TLR4 complex was successfully done, based on the calculated average temperature of 300 K (range: 298–302 K), average pressure of 0.953073 bar (range:  − 200 to + 200 bar), average potential energy of  − 2695122.552 kJ/mol (range:  − 270247 to  − 2687123 kJ/mol) and average density of 1009 kg/m^3^ (range: 1006–1012) during 100 ns. Based on the total energy analysis, the energy values for the vaccine-ligand complex were converged, averaged  − 2243714.486 kJ/mol. To understand the mobile nature of complex, the radius of gyration (Rg) was calculated, showing a constant rate during simulation for the receptor protein (3.195) and designed peptide (2.194), which means molecular stability during the process (Supplementary Figures). The RMSD plot of the human TLR-4 indicated values between 0.11 to 0.40 nm (Fig. 6A). The vaccine’s RMSD plot demonstrated an upward trend initially, with a peak at 0.84 nm after 46 ns of simulation, then reaching a relative plateau at 0.7 nm after 60 ns, due to the flexible nature of the receptor (Fig. 6B). In the following, the root mean square fluctuation (RMSF) values were plotted for both the receptor and *Leish*-App peptide. In the receptor protein, two peaks at the terminal regions indicated the highly-flexible parts (Fig. 6C). Also, due to the helical peptide structure, terminally-located residues were more flexible, while 5–25 residues showed more robust, hydrogenic bonds, hence lower fluctuation (Fig. 6D). The mean RMSF values for both peptide and protein were estimated to be 0.15 and 0.11, respectively. Hydrogen bond analysis, important for structural dynamics and complex stability, was done using gmx hbond order. The results showed that the peptide establishes stable hydrogen bonds (average: 9.4 bonds, up to 16 bonds) with the receptor during stimulation, leading to strong protein-peptide complex (Supplementary Figures).

### Vaccine safety and in silico cloning

**Figure 7:**
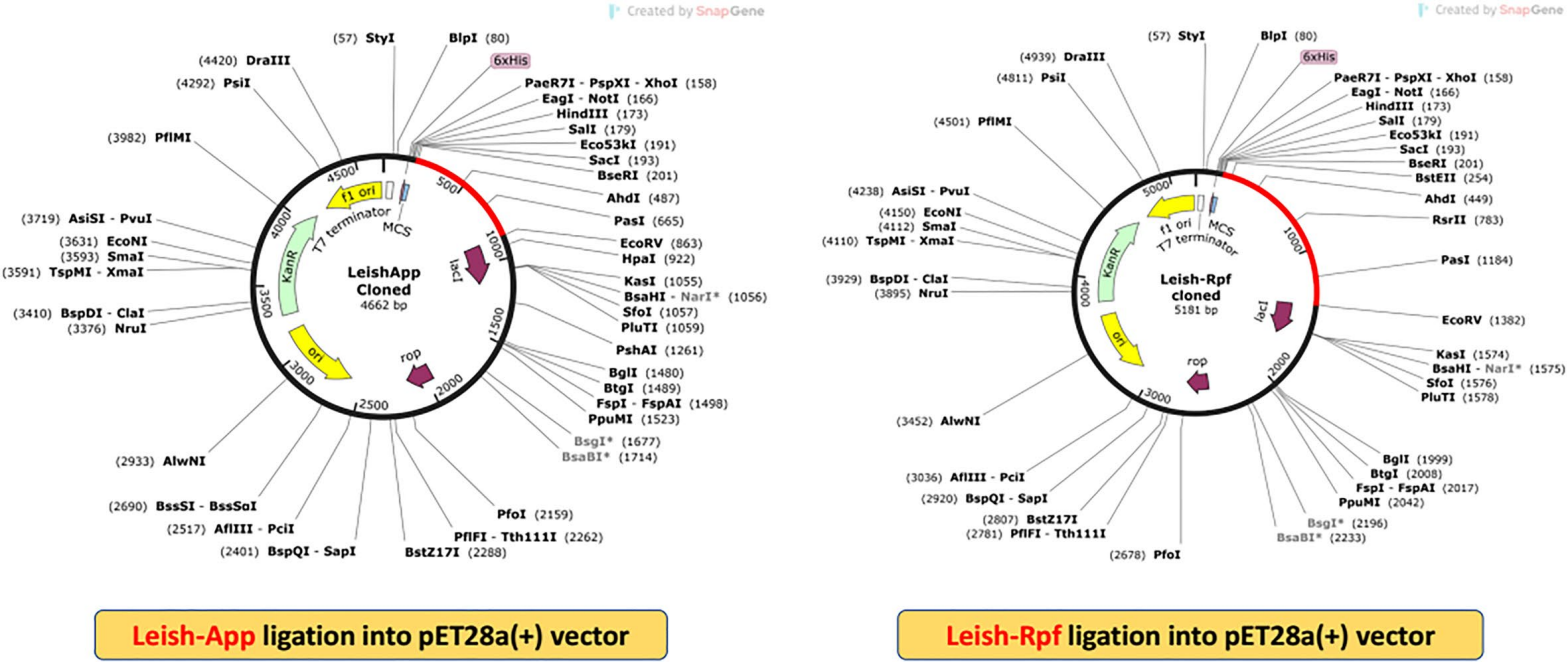
*In-silico* cloning of the Leish-*App* and Leish-*Rpf* vaccine sequences into the pET28a( +) vector using SnapGene 6.2.2.

Based on the BLASTp tool output of the UniProtKB server, none of the selected vaccine sequences (Leish-*App* and Leish-*Rpf*) had homology with the human proteome, hence considered to be safe for administration. Since the cutting sites of Eco53KI and EcoRV were absent in both vaccine sequences, hence they were embedded at the N- and C-terminal of sequences, along with the Shine-Dalgarno sequence and start/stop codons. The reverse translated “Leish-*App*” and “Leish-*Rpf*” vaccine models were further codon optimized for enhanced expression in *E. coli* K12 strain using JCat web tool. A similar GC content was estimated in Leish-*App* and Leish-*Rpf* vaccine candidates, with 59.32% and 58.90, respectively, and the CAI values for both models were calculated to be 1. Ultimately, the final vaccine sequences were successfully cloned into the pET28a( +) plasmid using SnapGene v6.2.2. software. The total length of the formed clones was 4662 bp (Leish-*App*) and 5181 bp (Leish-*Rpf*) (Fig. 7).

## Discussion

Leishmaniasis is highly prevalent in subtropical countries, with considerable burden every year^19^. Since vector and reservoir control strategies are not fully effective, development of efficacious vaccines seems to be an appropriate and sole preventive to reduce disease expansion^20^. Historically, leishmanization was shown to be the most effective strategy to control leishmaniasis^21,22^. Second and third generation vaccines such as the Leishmune (fucose-mannose ligand)^23^, Leish-Tec (Adenoviral-expressing *L. donovani* A2 protein)^24^, gp63 DNA vaccine^25^ as well as Leish-111f. recombinant vaccine (LeIF, LmSTI-1 and TSA) in Brazil, Peru and USA^26^ are promising examples of efficient immunization against leishmaniasis. Peptide-based immunogenic constructs, discovered by a number of in silico (B- and T-cell epitope prediction, protein localization, conservation analysis, etc.) and in vitro methods (Bio-panning assay, peptide-binding assay), constitute the next-generation vaccines, capable to efficiently target the appropriate classes of immune cells, in order to yield more effective immunity against a given pathogen^27^. This approach seems to be highly stable and reproducible, with decreased antigen complexity and lower costs for mass production. In this sense, the KSAC polyprotein has shown protection against *L. major* lesion formation in BALB/c mice^28^.

As mentioned earlier, *Leishmania* possesses a diverse array of antigenic compounds, which might be employed in rational vaccine design studies^29^. After preliminary in silico screening of 18 *L. major* vaccine candidate antigens, six proteins (gp46, CatL, CatB, H2A, grp78 and STI-1) were shown as the best targets for vaccination, since they had higher antigenicity scores and/or signal peptide/transmembrane domain. The surface glycoprotein gp46 is expressed in most parasite species and cells with reactivity against this protein were shown to produce a higher level of IFN-γ, conferring natural immunity^30^. It has, also, been demonstrated to elicit significant immune responses against *L. amazonensis*^31^ and *L. major*^32^ experimentally. Also, STI-1 is a novel antigen with copies of tetratricopeptide consensus motif with constitutive expression in both amastigote and promastigote stages^33^. Previously several studies have demonstrated its role as a potent vaccine candidate in immunization studies against CL^34–36^. The chaperonic protein, grp78, is functionally associated with the members of the highly-conserved 70 kDa stress protein family^8^. It has been shown that recombinant grp78 or its DNA induce protection in BALB/c, C57BL/6 and C3H/He mice against *L. major* infection^37^. Histones are evolutionarily-conserved, DNA-associated proteins, among which core histones can efficiently kill some *Leishmania* species, including *L. major* as antimicrobial peptides^29^. Cysteine proteases are key factors in extracellular matrix degradation and parasite propagation within host tissues^38^; among these, CatL and CatB molecules, found in all *Leishmania* species, play a pivotal role in parasite virulence^39^. Two expression plasmid containing cysteine proteinase genes (cpa and cpb) provided durable protection against CL through IFN-γ induction^40^. Based on the immunogenic capacity of these selected proteins, immunodominant HLA-binding epitopes were spotted in gp46, CatL, CatB, H2A, grp78 and STI-1 proteins of *L. major* and the potency of different designed MEVCs were evaluated using comprehensive in silico approaches.

At the first step, extensive epitope prediction and screening was performed using HLA reference set alleles, provided by the IEDB server. Herein, four T-cell-based MEVCs were designed and engineered using HLA-specific, IFN-γ Inducing epitopic fragments of six *L. major* antigens (gp46, CatL, CatB, H2A, grp78 and STI-1). Notably, B-cell epitopes were excluded from the prediction step, since humoral immune responses and higher levels of specific antibodies fail to provide protection against *Leishmania* infections, and may be strong predictors of parasite persistence^41^. Additionally, those CD_8_^+^, IFN-*γ* inducing T cell epitopes were predicted which are involved in parasite killing and lesion recovery^41^. A crucial part in the multi-epitope vaccine design is the utilization of appropriate linkers. Linkers or spacers are responsible for the flexibility of the polyprotein, its proper folding and segregation of functional domains, rendering a more stable protein structure^42^. The “AAY” linker, used to connect CTL epitopes in the current study, increases epitope partitioning and presentation through making the C-termini of CTL epitopes more accessible for binding^43^. Also, a glycine-rich linker, “GPGPG”, not only enhances the construct solubility, but also provides flexibility, high accessibility and free activity for adjacent domains. Of note, these epitopes are capable to induce HTL responses^44–46^. Consistent with our study, Khatoon et al*.*^47^ and Shams et al*.*^48^, also, employed AAY and GPGPG spacers to adjoin the epitope fragments of multi-epitope to be used against visceral leishmaniasis. In addition, “GDGDG” is known as a flexible linker, previously being used to link both CTL and HTL epitopes and design a multi-epitope candidate against *L. major*^49^. In the following, we used a rigid, non-flexible linker, “EAAAK”, after the adjuvant sequence, in order to prevent possible interaction with the rest of the vaccine sequence and prevent the formation of neo-epitopes^50^. Adjuvants are known as innate immune boosters or catalysts, and their utilization is implicated by the type of immune response required to be elicited. There are a wide range of genetic adjuvants, which can be embedded in the MEVCs^51^; in the present study, three different adjuvants were employed, such as a TLR-4 agonist (APPHALS), human IFN-*γ* and *M. tuberculosis* RpfE. The RS-09 synthetic peptide is an excellent adjuvant, previously used in many studies^52–54^. It allows the co-stimulation of CTL epitopes, providing a more robust immune response^55^. Moreover, another TLR-4 agonist, RpfE adjuvant, promotes dendritic cell maturation and enhances differentiation of naïve CD_4_^+^ T-cells toward Th1 population^56^. Also, the adjuvanticity of IFN-*γ* has been studied previously^57–59^.

The final stretch of four designed MEVCs was found to be 218 (Leish-*App*), 377 (Leish-*Ifn*), 366 (Leish-*Apfn*) and 391 (Leish-*Rpf*) residues. A good vaccine candidate should possess proper physico-chemical properties during production phase, along with strong immune stimulation. On this basis, the ProtParam web server was used for physico-chemical evaluation. The output showed that the largest MW belonged to Leish-*Ifn* (40.25 kDa), followed by Leish-*Apfn* (39.37 kDa) and Leish-*Rpf* (38.95 kDa), while the lowest MW was associated with the Leish-*App* candidate, with 21.59 kDa, rendering a more convenient extraction. With the exception of Leish-*App*, other designed vaccine candidates were shown to be highly hydrophilic in nature, based on a negative GRAVY score. Moreover, all candidates had stable molecular structure (instability index < 40) and were highly thermotolerant (high aliphatic index value). All candidates possessed no allergenic traits, through investigation by AllergenFP v1.0 and AllerTOP v2.0 servers, while two constructs (Leish-*App* and Leish-*Rpf*) demonstrated the highest antigenicity and solubility scores. Random coils were the most plentiful secondary structures in three candidates, except of Leish-*Apfn*, consistent with previous multi-epitope vaccine studies against *L. major* by Rabienia et al.^49^and against *L. donovani* by Khatoon et al*.*^47^.

In the present study, we selected two potent MEVCs, based on their immune profile shown by C-ImmSim web server. Actually, Leish-*App* and Leish-*Rpf* demonstrated extensive cell-mediate immune induction, in comparison with two other constructs, as evidenced by highest numbers of duplicating and active Th cells, specific memory T cells and elicited Th1-type cytokines (IFN-γ and IL-2). In comparison with *Leishmania* gp46 and LeIF protein sequences, as positive controls in immune simulation step, both vaccine candidates had similar expression of IFN-γ, as a representative of Th1-biased, protective response. Such immune excitement by these two vaccine candidates may partly arise from the immunogenic nature of the used adjuvants as potent TLR-4 agonists, *i.e.*, RS-09 synthetic peptide and RpfE of *M. tuberculosis*. Next, the 3D structure of two selected MEVCs were further refined using GalaxyRefine server for global and regional structural relaxations. In the following, the Ramachandran plot analysis by the PROCHECK tool showed satisfactory results, with most residues tightly clustered in the most favored and additional allowed regions. Next, a protein–protein docking analysis was performed using ClusPro 2.0 server. Based on the member density and the estimated binding scores, Leish-*App*/TLR-4 docked complex possessed better interactions than Leish-*Rpf*/TLR-4. Accordingly, the MD simulation study was performed on *Leish*-App/TLR-4 complex. Deviations in the polypeptide chain structure, compared to the reference one (the structure after molecular docking) and during simulation were shown as RMSD plots. Our results revealed the stability of the vaccine-TLR4 complex during the MD simulation study. Also, the RMSF analysis was done to determine the most flexible residues; generally, most parts were relatively rigid and stable, particularly those residues located internally. Moreover, hydrogen bond calculation between the vaccine peptide and receptor protein demonstrated highly stable complex, with an average of 9.4 hydrogen bonds. It was noteworthy that both candidates showed no homology to the human proteome, hence were considered to be safe for human use. Ultimately, improvements in transcriptional and translational efficiency was done using codon optimization, directed towards high-level expression yield of the recombinant proteins. For this purpose, total GC content and CAI value of Leish-*App* and Leish-*Rpf* DNA sequences were analyzed against *E. coli* (strain K12) using JCat server. In the final step, two proposed MEVCs against *L. major* were successfully ligated into the pET28a( +) vector, being prepared for subsequent vaccine production.

In the literature, some studies have performed immunoinformatics-based predictions to design, engineer and evaluate different MEVCs against leishmaniases. In a study by Hashemzadeh et al*.* (2019), three *L. infantum* proteins, gp63, KMP-11 and HSP-70 were targeted for B- and T-cell epitope prediction and a 45.9 kDa polyprotein was designed using GGGGS and GSGSGS linkers, connected to two adjuvants (*M. tuberculosis* RpfE and RpfBG G5 domain)^60^. However, this study lacked the secondary structure analysis, molecular docking and vaccine immune profile evaluation. Rabienia et al.^49^ designed a 27.17 kDa MEVC using B-cell, T-cell and IFN-γ Inducing epitopes of *L. major* HASPB and KMP-11, in association with GDGDG linker and profilin as adjuvant; they showed proper and stable interaction between the vaccine model and TLR-11 receptor, without the prediction of immune stimulation. Another study by Yadav et al.^61^ designed a 71 kDa, stable and hydrophilic multi-component vaccine candidate using B- and T-cell epitopes derived from three *L. donovani* HyP proteins, prevailed by random coils, using AAY and KK spacers. Ropon-Palacios et al.^62^ investigated the *in-silico* binding of a novel multi-component vaccine (32.5 kDa), designed by 4 conserved epitopes from the Latin American species such as *L. braziliensis*, *L. mexicana*, *L. panamensis* and *L. guyanensis*, with TLR4/MD2 receptor complex, showing a stable interaction. In our study, four designed MEVCs were initially screened in terms of basic physico-chemical, antigenicity, allergenicity, solubility and immune profile characteristics, then two potent candidates were further evaluated regarding 3D structure refinement, molecular docking, vaccine safety and in silico cloning.

## Conclusion

The safety and rational design nature of the multi-epitope vaccines have led researchers towards engineering more robust, stable and efficient vaccine candidates against many pathogens and cancers. Hence this novel field of immunoinformatics saves time and experimental resources and deserves further exploration. The aim of the present study was to design four novel MEVCs against CL through initial screening of 18 *L. major* vaccine candidate antigens (H1, H2A, H2B and H4, HSP60, HSP70, HSP83 (HSP90), HSP100, rP0, KMP11, TSA, LeIF, LACK, STI-1, gp46, CatL, CatB and grp78), and by arranging different linkers (GDGDG, GPGPG, AAY, EAAAK) and/or adjuvants (RS-09 synthetic peptide, RpfE and human IFN-γ) among IFN-γ Inducing T-cell epitopes. As a final word, two selected vaccine models in the present study (Leish-*App* and Leish-*Rpf*) demonstrated antigenic, allergenic, solubility, safety and physico-chemical properties. Moreover, adequate binding score and members were predicted between the vaccine candidates and TLR-4 receptor, along with robust cell-mediated immune stimulation. It is finally noteworthy that in vitro and in vivo experiments are demanded to validate the efficacy of the proposed MEVCs.

## Methods

**Figure 8:**
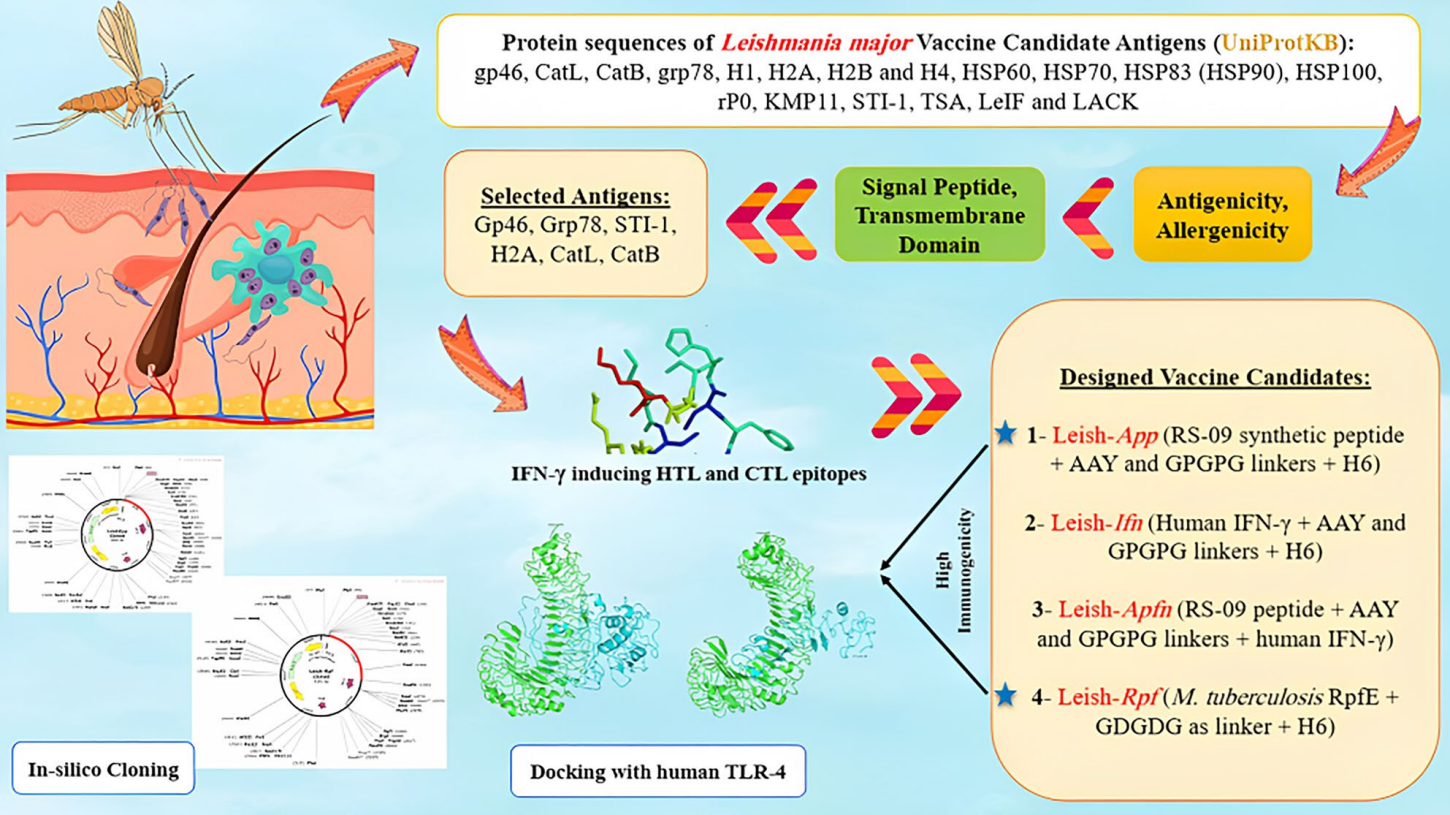
Schematic representation of the MEVC design from selected *L. major* vaccine candidate antigens and their evaluation. Leish-*App* and Leish-*Rpf* were selected as two immunogenic candidates.

A schematic representation of the antigen selection and MEVC design is provided in Fig. 8.

### Antigen selection and amino acid sequence retrieval

A preliminary study was done on 18 *L. major* vaccine candidate antigens explored in leishmaniasis vaccination studies, including histone proteins H1, H2A, H2B and H4, heat shock proteins HSP60, HSP70, HSP83 (HSP90), HSP100, ribosomal protein P0 (rP0), kinetoplast membrane protein 11 (KMP11), thiol-specific antioxidant (TSA), *Leishmania* elongation initiation factor (LeIF), *Leishmania* activated C-kinase antigen (LACK), STI-1, gp46, CatL, CatB and grp78 (data not shown); among these six antigens were shown to be highly antigenic (STI-1 and H2A) or possessed signal peptide and transmembrane domains (gp46, CatL, CatB, grp78), and they were included in the present study to design different antigenic MEVCs against CL. The amino acid sequence of six selected *L. major* proteins was retrieved through a leading high quality, comprehensive and freely-accessible resource of protein sequences and functional information, UniProt Knowledge Base^63^, available at https://www.uniprot.org/, with the following accession numbers: Q4Q6B6 (**gp46**), P90627 (**CatB**), Q4QI62 (**CatL**), Q4Q8E6 (**grp78**), Q4QEG6 (**H2A**) and Q4Q271 (**STI-1**).

### Prediction of HLA-restricted IFN-γ Inducing Epitopes in six selected *L. major* antigens

Major histocompatibility complex class-II (MHC-II) binders, the so-called HTL epitopes, were predicted using MHC-II epitope prediction of the IEDB web server using recommended method (http://tools.iedb.org/mhcii/), “Human” as the target host and “HLA reference set alleles” option (Population coverage over 97%)^64^. The top-ten high-ranked epitopes possessing lower percentile rank were then screened regarding antigenicity and IFN-*γ* inducing epitopes, by using VaxiJen v2.0 (http://www.ddg-pharmfac.net/vaxijen/VaxiJen/VaxiJen.html) and IFNepitope (http://crdd.osdd.net/raghava/ifnepitope/) online tools, respectively. The latter employs a dataset of MHC-II-binding IFN-*γ*-inducers and non-inducers and the most accuracy can be reached by selecting hybrid model (> 81.39%), as we did in the current prediction (Supplementary Table 1). Those 9–10-mer CTL epitopes (MHC-I binders) specific to humans were predicted using IEDB MHC-I epitope prediction tool, available at http://tools.iedb.org/mhci/, using IEDB recommended method 2020.09 (NetMHCpan EL 4.1)^64^, with the selection of reference HLA allele set, including 16 class A alleles (01:01, 02:01, 02:03, 02:06, 03:01, 11:01, 23:01, 24:02, 26:01, 30:01, 30:02, 31:01, 32:01, 33:01, 68:01 and 68:02) and 11 class B alleles (07:02, 08:01, 15:01, 35:01, 40:01, 44:02, 44:03, 51:01, 53:01, 57 l:01 and 58:01)^65^. The top-ten high-affinity epitopes having a percentile rank < 1 were screened in terms of immunogenicity and IFN-*γ* induction using the immunogenicity tool in the IEDB server (http://tools.iedb.org/immunogenicity/) and IFNepitope server (Supplementary Table 2). All potent human CTL and HTL epitopes were, also, screened in terms of toxicity and allergenicity, using ToxinPred (http://crdd.osdd.net/raghava/toxinpred/) and AllergenFP v1.0 (https://www.ddg-pharmfac.net/AllergenFP/) web servers, respectively.

### Design and assemblage of potent MEVCs

Accurately predicted and strictly screened human CTL (N = 4) and HTL (N = 7) epitopes were involved in the MEVC design process. In this study, three different adjuvants were used in designing four potent vaccine candidates, including a Toll-Like Receptor 4 (TLR-4) agonist (RS-09 peptide), human IFN-*γ* and *Mycobacterium tuberculosis* resuscitation-promoting factor E (RpfE). Moreover, different linkers such as “EAAAK”, “GDGDG”, “AAY’ and “GPGPG” were employed to connect adjuvants and/or immunodominant epitopes and design different MEVCs. Of note, a novel double-adjuvant vaccine candidate was introduced in the present study, incorporating RS-09 peptide and human IFN-*γ* at the N- and C-termini of the vaccine sequence, respectively.

### Evaluation of allergenicity, antigenicity, solubility and physico-chemical properties of MEVCs

Two web servers were used to evaluate allergenicity, including AllergenFP v1.0 and AllerTOP v2.0 (https://www.ddg-pharmfac.net/AllerTOP/). The latter performs an 85.3% prediction through transforming the amino acid sequences into the integral vectors with equivalent lengths, based on auto cross covariance (ACC)^66^. Also, a novel alignment-independent, descriptor-based fingerprint method is used by AllergenFP v1.0 using physico-chemical and structural properties, making a prediction with 88% accuracy^67^. In the following, VaxiJen server was used to evaluate antigenicity of different MEVCs; which performs on the basis of ACC transformation of the sequences into uniform vectors, being truly depended on the proteins chemical nature and make predictions with 70–89% accuracy^68^. The Protein-Sol server, available at https://protein-sol.manchester.ac.uk/, was utilized for the prediction of protein solubility, so that protein scores over 0.45 are considered as soluble^69^. In the next step, major physico-chemical characteristics of the engineered MEVCs were predicted using ExPASy ProtParam web tool, available at https://web.expasy.org/protparam/. The server predicts several parameters, comprising molecular weight (MW), theoretical isoelectric point (pI), amino acid composition, in vitro and in vivo protein half-life, aliphatic index, instability index and the grand average of hydropathicity (GRAVY) score^70^.

### Secondary and tertiary structure prediction for MEVCs

Secondary structure of MEVCs was predicted using Garnier–Osguthorpe–Robson IV (GOR IV) server (https://npsa-prabi.ibcp.fr/NPSA/npsa_gor4.html). This server predicts the number and percentage of residues in different secondary structures, including alpha helix, extended strand and random coil^71^. In the following, the Iterative Threading ASSEmbly Refinement (I-TASSER) server, available at https://zhanggroup.org/I-TASSER/, was used for three-dimensional (3D) modelling for the submitted MEVCs. It employs different 3D templates for the homology modelling of the input sequence, and finally provides tope five 3D models, having different C-scores. A C-score is an index for confidence of prediction, ranging between  − 5 and 2, so that those models having higher C-scores can be considered as more reliable^72^.

### Immune simulation profile of different MEVCs

The virtual immune simulation process elicited by four designed MEVs was predicted using C-ImmSim online server, available at http://150.146.2.1/C-IMMSIM/index.php. “These predictions are premised on PSSM for machine learning methods. The output implies to three stimulated regions including bone marrow, thymus and lymph node^73^. This computer-aided simulation was accomplished using default parameters with random seed 12,345, simulation volume 10 and simulation steps 100″. Based on the results, two highly immunogenic MEVCs were chosen for further analysis. For cytokine expression comparison, *L. major* gp46 (ID: Q4Q6B6) and LeIF (ID: W5XL77) protein sequences were selected from UniProt, as positive controls.

### Tertiary model refinement and validations

The GalaxyRefine web server was employed to refine the 3D model of the best immunogenic MEVCs through mild/aggressive quality improvement. Several parameters are provided as output, encompassing global distance test-high accuracy (GDT-HA), root mean square deviation (RMSD), MolProbity, Clash score, Poor rotamers and Rama favored^74^. In the following, the quality of the rehashing process was validated, in comparison with crude models, using Ramachandran plot analysis; for this aim, the PROCHECK tool of the SAVES v6.0 server was used. “The server evaluates the stereochemical quality of a protein structure, producing a number of PostScript plots analyzing its overall and regional qualities”^75^.

### Molecular interaction between selected MEVCs and TLR-4

For this aim, the 3D structure of human TLR-4/MD2 molecule (Accession No.: 3FXI) was retrieved via the PDB database of Research Collaboratory for Structural Bioinformatics (RCSB) (https://www.rcsb.org). Next, ClusPro 2.0 protein–protein docking web server, available at https://cluspro.bu.edu, was used to predict the affinity between the amino acid sequences of the best immunogenic MEVCs designed and engineered in the present study (as ligand) and human TLR-4 (as receptor) using default settings^76^. “The output of the server is provided as a top-rank cluster, among which the best docking pose is selected for visualization”^77^.

### Molecular dynamics (MD) simulations

The MD study was carried out for *Leish*-App and human TLR-4 complex, using GROMCAS 2020 software. The biomolecular properties of *Leishmania*-associated multi-epitope vaccine construct complexed with TLR-4 were given to the software using pdb2gmx module. Next, the OPLS-AA/L all atom force field was selected, the complex was confined in a rectangular box, then dissolved in water. Using the solvate and editconf modules, the size of the system was determined based on the biomolecule extent, and the thickness of the water layer above and below the protein was considered to be 10 Å. The PME (Particle-mesh-Ewald) method was used to neutralize the system for long-range electrostatic interactions. Also, at this stage, KCl salt ions at a near physiological concentration (0.15 M) were added to the system for electrical neutralization. The number of Cl^−^ and K^+^ ions was automatically determined by the ion accessible volume (V) and total system charge (Qsys). In the following, energy minimization (EM) was done in order to allow the system to reach the lowest possible energy and the most stable state possible prior to starting dynamics. This aim was done by PEM decreasing slope method and continued until the maximum force was smaller than 1000 kJ/mol/nm. Next, to prevent the system to crash, equilibration was applied between the solvent and the ions around the protein. Equilibration is often done in two steps. First, it takes place under a NVT (constant number of particles, volume and temperature) set, in which the temperature of the system must reach a plateau at the desired value. After stabilizing the temperature, pressure equilibration was performed under an NPT set using Parrinello-Rahman barostat, where the number of particles, pressure, and temperature are all constant. Finally, the simulation step was performed for 100 ns for the system containing the docked complex and the simulation quality was evaluated using the gmx check tool. The analysis was done with the gmx Energy tool.

### Safety prediction, codon optimization and in silico cloning

For this aim, the BLAST online tool of the UniProtKB server (https://www.uniprot.org/blast) was utilized, in order to discriminate likely similar regions between selected vaccine candidate sequences and the respective organism. In this study, the human proteome was defined as target and identity rates over 35% mean homologous proteins with the human proteome^78^.

Efficient protein expression in the host bacterium, *Escherichia coli*, is a crucial step for subunit vaccine production. Accordingly, reverse translation of the selected MEVCs was done using the reverse translate tool of the Sequence Manipulation Suite (https://www.bioinformatics.org/sms2/rev_trans.html), followed by codon optimization using JCat server (http://www.jcat.de/). The JCat server assesses several important properties of the DNA sequence, such as GC content and codon adaptation index (CAI), which are significantly implicated in the expression of chimeric proteins in their respective hosts. Hence, in the present study, codon optimization was performed based on *E. coli* K12 strain^79^. Next, NEBcutter 2 server (https://nc2.neb.com/NEBcutter2/) was employed to evaluate the presence of cutting sites of different commercially available restriction enzymes within codon-adapted vaccine sequences. Ultimately, the cutting sites of two restriction enzymes, *Eco53KI* and *EcoRV,* were added to the 5′- and 3′-OH of the codon optimized vaccine sequence. Moreover, the Shine-Dalgarno sequence (AGGAGG) was added before the start codon to improve the yield of expression. The *in-silico* cloning process of the selected final multimeric vaccine models was accomplished using SnapGene v6.2.2. standalone software (from Insightful Science; available at https://www.snapgene.com).

### Supplementary Information

Supplementary Figures.

Supplementary Table S1.

Supplementary Table S2.

## Data Availability

All data generated or analysed during this study are included in this published article (and its Supplementary Information files).
