# Supplementary material for: Engineering and design of promising T-cell-based multi-epitope vaccine candidates against leishmaniasis
Source: Sci Rep. 2023 Nov 8;13:19421. doi: 10.1038/s41598-023-46408-1 (PMC10632461; doi:10.1038/s41598-023-46408-1)
Supplement: Supplementary file 1 — Supplementary Figures. [file 41598_2023_46408_MOESM1_ESM.pdf]

Supplementary Figures

Leish-*App* and Leish-*Rpf* as two promising T-cell-based Multi-Epitope Vaccine Candidates Against Leishmaniasis

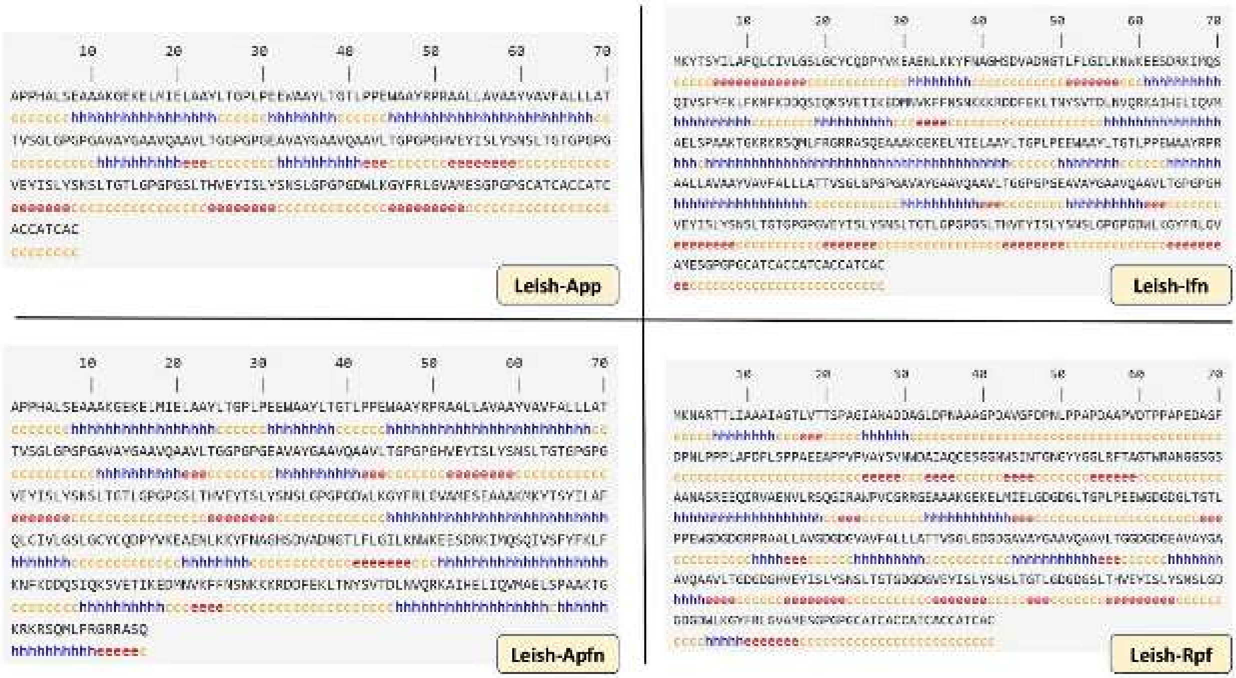

Supplementary Figure 1. Secondary structure prediction using GOR IV server for 4 deigned MEVCs.

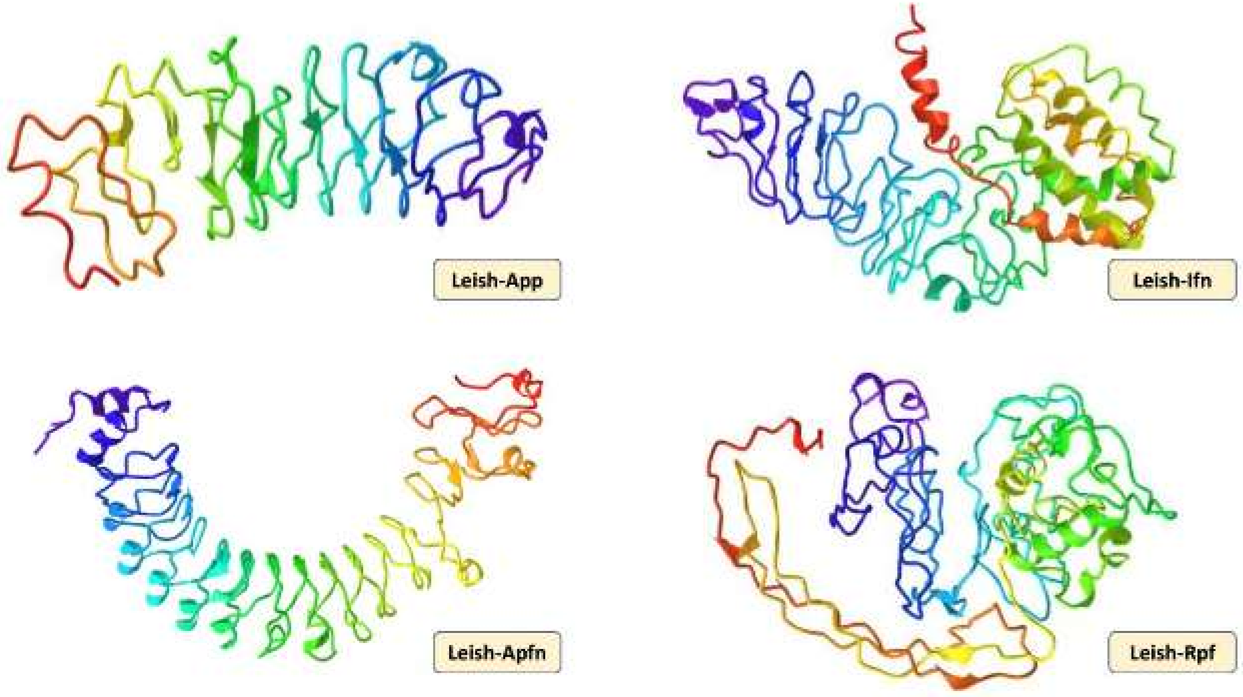

Supplementary Figure 2. Homology modelling using I-TASSER web server for 4 deigned MEVCs.

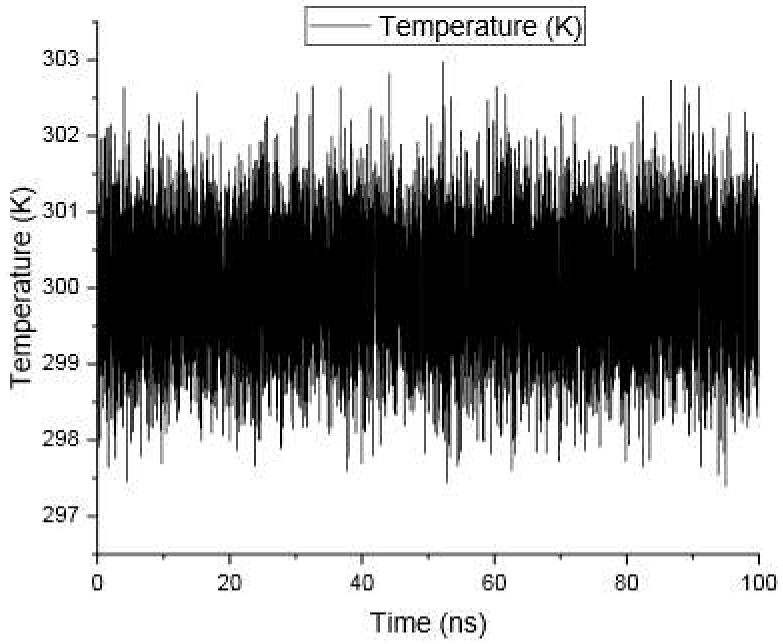

Supplementary Figure 3. The trend of temperature during MD simulation.

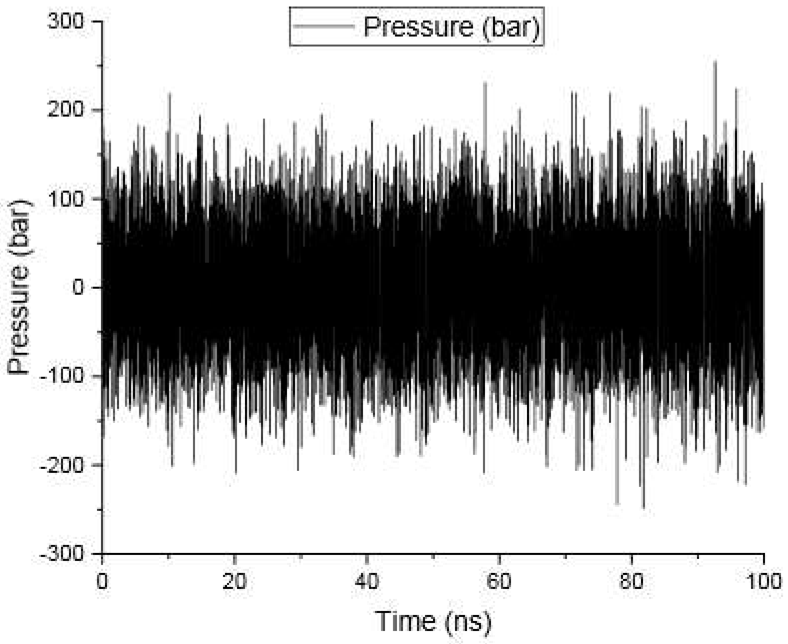

Supplementary Figure 4. The trend of pressure during MD simulation.

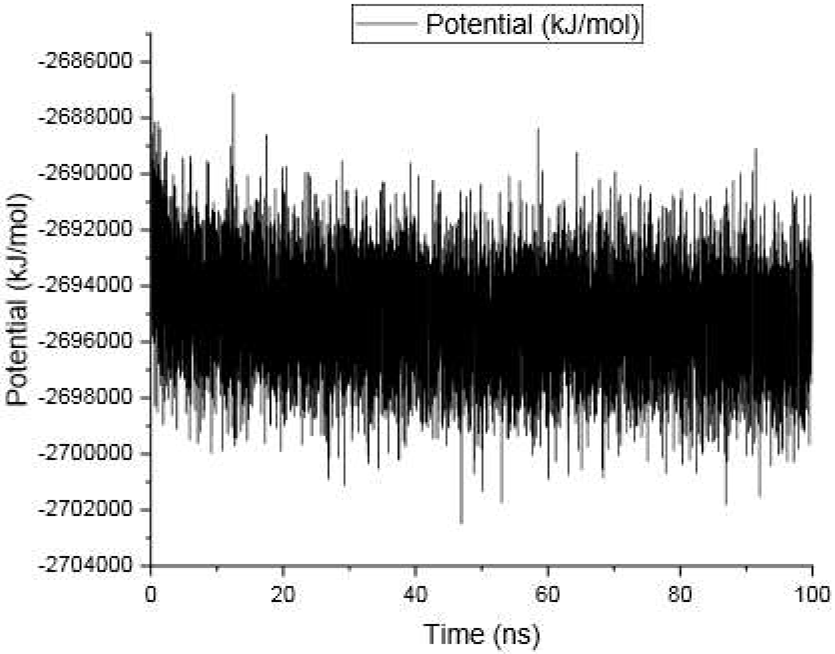

Supplementary Figure 5. The trend of potential energy during MD simulation.

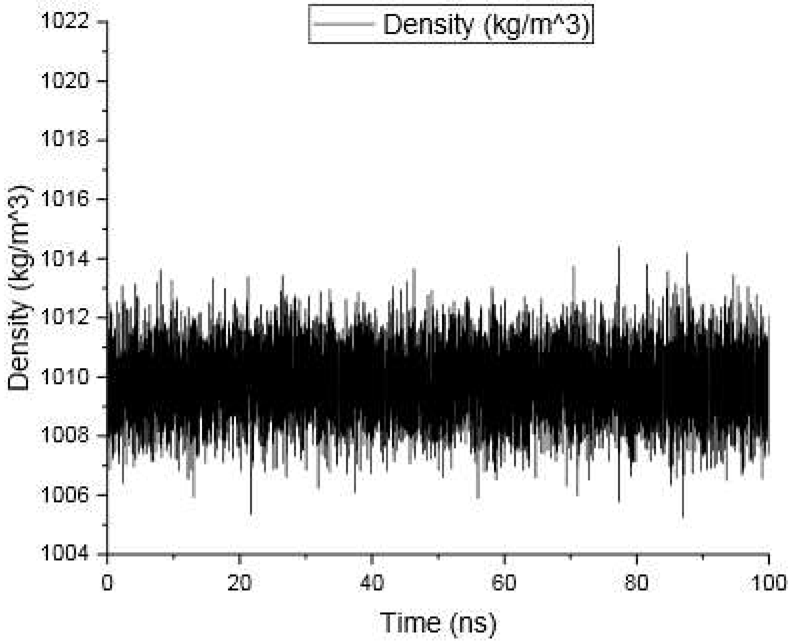

Supplementary Figure 6. The trend of density during MD simulation.

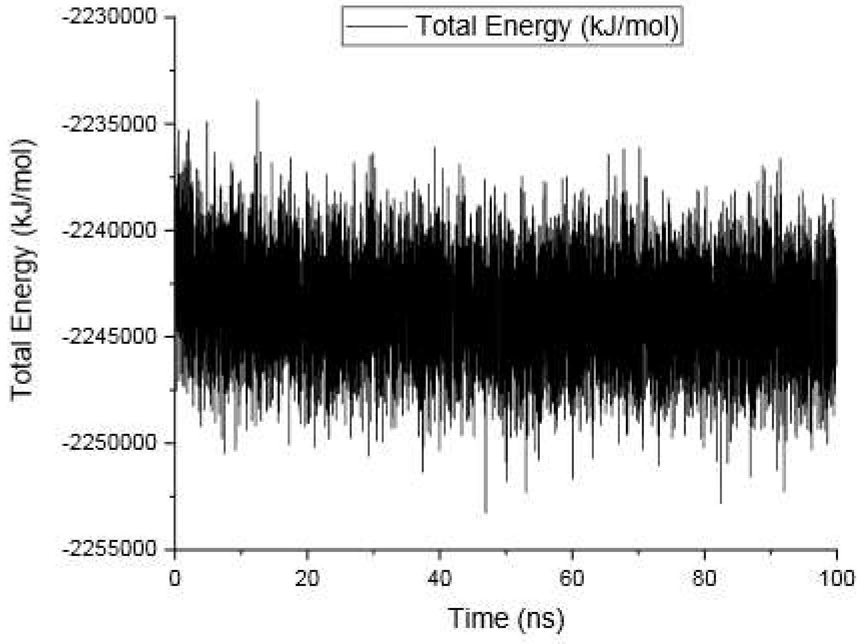

Supplementary Figure 7. The trend of total energy during MD simulation.

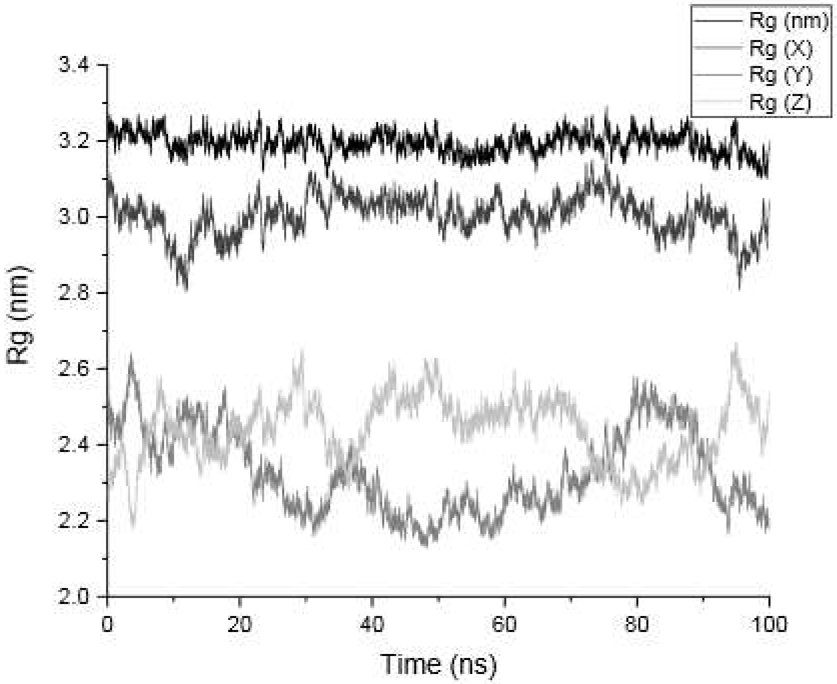

Supplementary Figure 8. Values of total rotation radius and rotation radius in X, Y and Z axes of the protein during 100 nanoseconds of simulation in nanometers

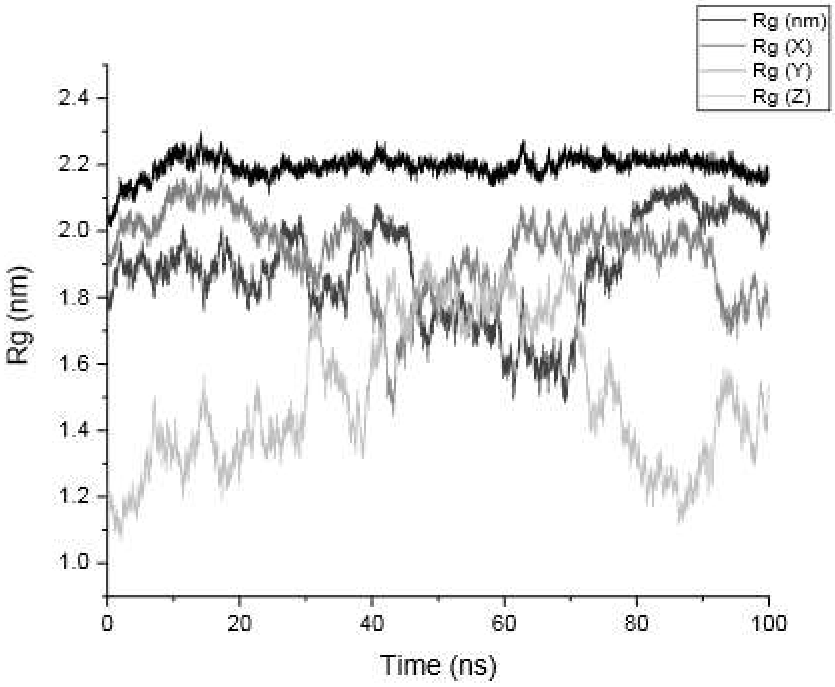

Supplementary Figure 9. Total radius of gyration and radius of gyration in the X, Y and Z axes of the peptide during 100 nanoseconds of simulation in nanometers

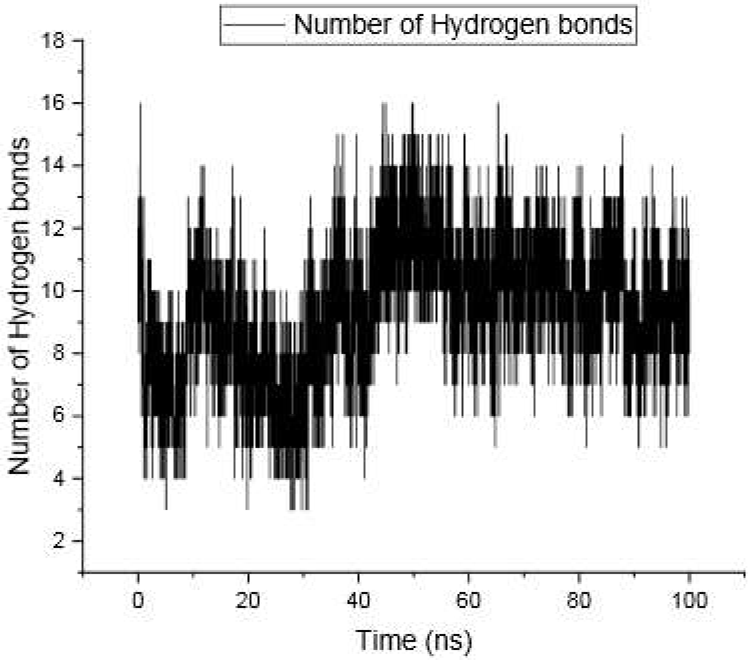

Supplementary Figure 10. The number of hydrogen bonds per 100 ns of MD simulation.

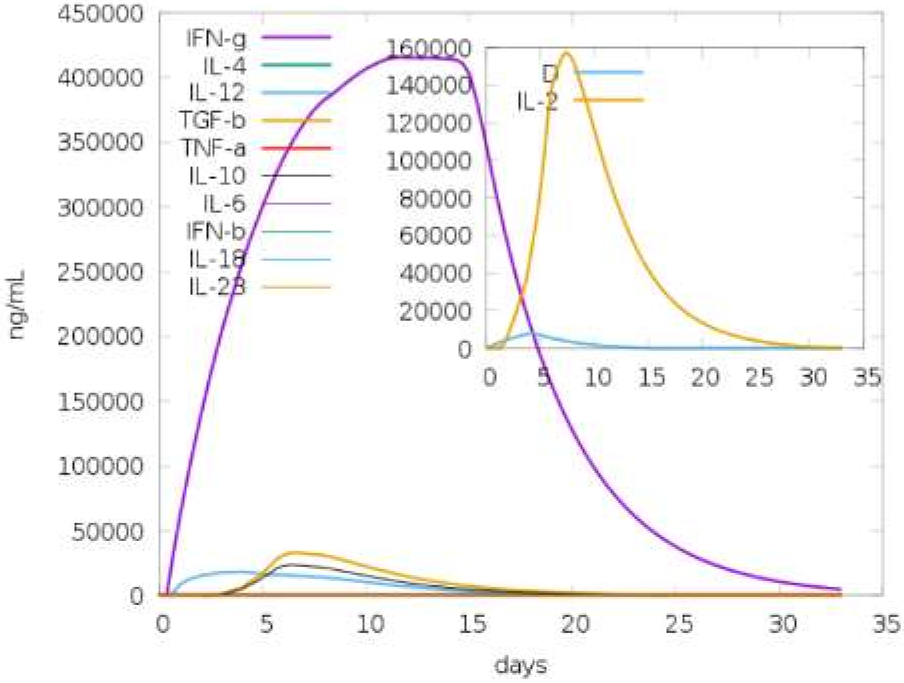

Supplementary Figure 11. Simulated cytokine profile upon *Leishmania* gp46 protein injection.

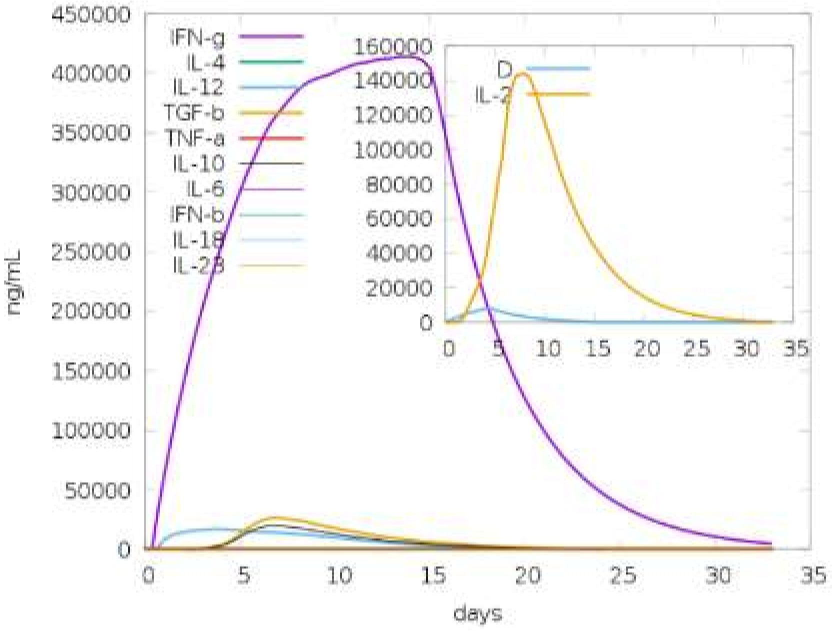

Supplementary Figure 12. Simulated cytokine profile upon *Leishmania* LeIF protein injection.
