# Supplementary material for: Engineering and design of promising T-cell-based multi-epitope vaccine candidates against leishmaniasis
Source: Sci Rep. 2023 Nov 8;13:19421. doi: 10.1038/s41598-023-46408-1 (PMC10632461; doi:10.1038/s41598-023-46408-1)
Supplement: Supplementary file 2 — Supplementary Table S1. [file 41598_2023_46408_MOESM2_ESM.docx]

**Supplementary Table S1**

**Leish-*App* and Leish-*Rpf* as two promising T-cell-based Multi-Epitope Vaccine Candidates Against Leishmaniasis**

**Supplementary Table S1.** Helper T-lymphocyte specific epitope prediction for six selected *L. major* vaccine candidate antigens and subsequent screening regarding antigenicity and IFN-γ induction

| **Protein** | **Allele** | **Start-End** | **HTL epitope** | **Method** | **Percentile rank** | **Antigenicity** | **IFN-γ inducing** | |
| --- | --- | --- | --- | --- | --- | --- | --- | --- |
|  |  |  |  |  |  |  | **Result** | **Score** |
| **Histone H2A** | HLA-DQA1*05:01/DQB1*02:01 | 42 - 56 | AALLEYLTTEVIELS | Consensus (comb.lib./smm/nn) | 0.14 | 0.1767 | Negative | 1 |
|  | HLA-DQA1*05:01/DQB1*02:01 | 41 - 55 | CAALLEYLTTEVIEL | Consensus (comb.lib./smm/nn) | 0.14 | 0.0573 | **Positive** | 0.4390 |
|  | HLA-DQA1*05:01/DQB1*02:01 | 43 – 57 | ALLEYLTTEVIELSG | Consensus (comb.lib./smm/nn) | 0.16 | 0.1851 | Negative | 1 |
|  | HLA-DQA1*05:01/DQB1*02:01 | 44 – 58 | LLEYLTTEVIELSGA | Consensus (comb.lib./smm/nn) | 0.17 | 0.0644 | Negative | 1 |
|  | HLA-DQA1*05:01/DQB1*02:01 | 45 – 59 | LEYLTTEVIELSGAA | Consensus (comb.lib./smm/nn) | 0.21 | 0.1995 | Negative | 1 |
|  | HLA-DQA1*01:02/DQB1*06:02 | 30 – 44 | KQRCGASAAIYCAAL | Consensus (comb.lib./smm/nn) | 0.56 | 0.8481 | Negative | 2 |
|  | HLA-DQA1*01:02/DQB1*06:02 | 31 – 45 | QRCGASAAIYCAALL | Consensus (comb.lib./smm/nn) | 0.56 | 0.9368 | Negative | 2 |
|  | HLA-DQA1*01:02/DQB1*06:02 | 32 – 46 | RCGASAAIYCAALLE | Consensus (comb.lib./smm/nn) | 0.58 | 0.8542 | Negative | 2 |
|  | HLA-DPA1*01:03/DPB1*04:01 | 42 – 56 | AALLEYLTTEVIELS | NetMHCIIpan | 0.95 | 0.1767 | Negative | 1 |
|  | HLA-DQA1*01:02/DQB1*06:02 | 29 - 43 | RKQRCGASAAIYCAA | Consensus (comb.lib./smm/nn) | 0.98 | 0.8097 | Negative | 2 |
| **Cathepsin L** | HLA-DPA1*01:03/DPB1*04:01 | 18 – 32 | HARFGITKFFDLSEA | NetMHCIIpan | 0.07 | -0.4792 | Negative | -0.3282 |
|  | HLA-DPA1*01:03/DPB1*04:01 | 17 – 31 | PHARFGITKFFDLSE | NetMHCIIpan | 0.1 | -0.4191 | Negative | -0.4224 |
|  | HLA-DPA1*01:03/DPB1*04:01 | 19 – 33 | ARFGITKFFDLSEAE | NetMHCIIpan | 0.12 | -0.7247 | Negative | -0.5605 |
|  | HLA-DPA1*01:03/DPB1*04:01 | 16 – 30 | NPHARFGITKFFDLS | NetMHCIIpan | 0.12 | -0.1432 | Negative | -0.3223 |
|  | HLA-DPA1*01:03/DPB1*04:01 | 15 – 29 | RNPHARFGITKFFDL | NetMHCIIpan | 0.18 | -0.0368 | Negative | -0.4443 |
|  | HLA-DRB1*13:02 | 16 – 30 | AFEWVLRNMNGTVFT | Consensus (smm/nn/sturniolo) | 0.22 | 0.3137 | Negative | 1 |
|  | HLA-DRB1*13:02 | 17 - 31 | FEWVLRNMNGTVFTE | Consensus (smm/nn/sturniolo) | 0.23 | 0.6973 | Negative | 1 |
|  | HLA-DRB1*13:02 | 15 – 29 | QAFEWVLRNMNGTVF | Consensus (smm/nn/sturniolo) | 0.23 | 0.2437 | Negative | 1 |
|  | HLA-DRB1*13:02 | 18 – 32 | EWVLRNMNGTVFTEK | Consensus (smm/nn/sturniolo) | 0.31 | 0.8513 | Negative | 1 |
|  | HLA-DPA1*01:03/DPB1*04:01 | 20 - 34 | RFGITKFFDLSEAEF | NetMHCIIpan | 0.32 | -0.7129 | Negative | -0.7353 |
| **Cathepsin B** | HLA-DRB1*01:01 | 13- 27 | AVFALLLATTVSGLY | Consensus (comb.lib./smm/nn) | 0.01 | 0.4028 | **Positive** | 0.7449 |
|  | HLA-DRB1*01:01 | 10 – 24 | CLVAVFALLLATTVS | Consensus (comb.lib./smm/nn) | 0.01 | 0.3264 | **Positive** | 0.4844 |
|  | HLA-DRB1*01:01 | 9 – 23 | LCLVAVFALLLATTV | Consensus (comb.lib./smm/nn) | 0.01 | 0.1915 | **Positive** | 0.5971 |
|  | HLA-DRB1*01:01 | 11 – 25 | LVAVFALLLATTVSG | Consensus (comb.lib./smm/nn) | 0.01 | 0.7546 | **Positive** | 0.5059 |
|  | HLA-DRB1*01:01 | 12 – 26 | VAVFALLLATTVSGL | Consensus (comb.lib./smm/nn) | 0.01 | 0.4544 | **Positive** | 0.8028 |
|  | HLA-DRB1*07:01 | 3 – 17 | LCLVAVFALLLATTV | Consensus (comb.lib./smm/nn) | 0.14 | 0.1915 | **Positive** | 0.5971 |
|  | HLA-DQA1*03:01/DQB1*03:02 | 15 – 29 | CGSCWAIAAVEAISD | Consensus (comb.lib./smm/nn) | 0.16 | -0.0601 | Negative | 1 |
|  | HLA-DRB1*01:01 | 14 – 28 | AKSALCLVAVFALLL | Consensus (comb.lib./smm/nn) | 0.16 | 0.1647 | Negative | 2 |
|  | HLA-DRB1*01:01 | 5 – 19 | ALCLVAVFALLLATT | Consensus (comb.lib./smm/nn) | 0.16 | 0.1066 | **Positive** | 0.4627 |
|  | HLA-DQA1*04:01/DQB1*04:02 | 8 - 22 | CGSCWAIAAVEAISD | Consensus (comb.lib./smm/nn) | 0.19 | -0.0601 | Negative | 1 |
| **Grp78** | HLA-DQA1*05:01/DQB1*03:01 | 38 – 52 | AVAYGAAVQAAVLTG | Consensus (comb.lib./smm/nn) | 0.09 | 1.0518 | **Positive** | 0.6594 |
|  | HLA-DQA1*05:01/DQB1*03:01 | 36 – 50 | DEAVAYGAAVQAAVL | Consensus (comb.lib./smm/nn) | 0.09 | 0.8555 | Negative | 1 |
|  | HLA-DQA1*05:01/DQB1*03:01 | 37 – 51 | EAVAYGAAVQAAVLT | Consensus (comb.lib./smm/nn) | 0.09 | 1.0503 | **Positive** | 0.6721 |
|  | HLA-DQA1*05:01/DQB1*03:01 | 35 – 49 | PDEAVAYGAAVQAAV | Consensus (comb.lib./smm/nn) | 0.09 | 0.8104 | Negative | 1 |
|  | HLA-DQA1*01:02/DQB1*06:02 | 11 – 25 | AVCLVSAILVVSAAA | Consensus (comb.lib./smm/nn) | 0.11 | 0.4557 | Negative | 2 |
|  | HLA-DQA1*01:02/DQB1*06:02 | 12 – 26 | VCLVSAILVVSAAAV | Consensus (comb.lib./smm/nn) | 0.11 | 0.3309 | Negative | 1 |
|  | HLA-DQA1*01:02/DQB1*06:02 | 13 – 27 | CLVSAILVVSAAAVP | Consensus (comb.lib./smm/nn) | 0.12 | 0.3708 | Negative | 1 |
|  | HLA-DQA1*01:02/DQB1*06:02 | 10 – 24 | MAVCLVSAILVVSAA | Consensus (comb.lib./smm/nn) | 0.12 | 0.5555 | Negative | 2 |
|  | HLA-DQA1*01:02/DQB1*06:02 | 9 – 23 | LMAVCLVSAILVVSA | Consensus (comb.lib./smm/nn) | 0.14 | 0.5999 | Negative | 2 |
|  | HLA-DQA1*01:02/DQB1*06:02 | 25 - 39 | INEPTAAAIAYGLNK | Consensus (comb.lib./smm/nn) | 0.16 | -0.0735 | Negative | 1 |
| **Gp46** | HLA-DRB1*01:01 | 136 – 150 | HVEYISLYSNSLTGT | Consensus (comb.lib./smm/nn) | 0.1 | 0.9449 | **Positive** | 1 |
|  | HLA-DRB1*01:01 | 135 – 149 | THVEYISLYSNSLTG | Consensus (comb.lib./smm/nn) | 0.1 | 0.9790 | Negative | -0.2514 |
|  | HLA-DRB1*01:01 | 137 - 151 | VEYISLYSNSLTGTL | Consensus (comb.lib./smm/nn) | 0.1 | 0.9697 | **Positive** | 1 |
|  | HLA-DRB1*04:05 | 134- 148 | LTHVEYISLYSNSLT | Consensus (smm/nn/sturniolo) | 0.12 | 0.5979 | Negative | -0.0820 |
|  | HLA-DRB1*04:05 | 135 – 149 | THVEYISLYSNSLTG | Consensus (smm/nn/sturniolo) | 0.15 | 0.9790 | Negative | -0.02514 |
|  | HLA-DRB1*01:01 | 134 – 148 | LTHVEYISLYSNSLT | Consensus (comb.lib./smm/nn) | 0.16 | 0.5979 | Negative | -0.0820 |
|  | HLA-DRB1*01:01 | 133 – 147 | SLTHVEYISLYSNSL | Consensus (comb.lib./smm/nn) | 0.16 | 0.4518 | **Positive** | 0.0511 |
|  | HLA-DRB1*04:05 | 136 – 150 | HVEYISLYSNSLTGT | Consensus (smm/nn/sturniolo) | 0.21 | 0.9449 | **Positive** | 1 |
|  | HLA-DRB1*09:01 | 152 – 166 | PPEWAKMKSAKWFLL | Consensus (comb.lib./smm/nn) | 0.3 | 0.4666 | Negative | -0.2440 |
|  | HLA-DRB1*04:05 | 133 - 147 | SLTHVEYISLYSNSL | Consensus (smm/nn/sturniolo) | 0.32 | 0.4518 | Negative | -0.1187 |
| **STI-1** | HLA-DPA1*02:01/DPB1*14:01 | 13 – 27 | KGYFRLGVAMESMVK | NetMHCIIpan | 0.02 | 0.5969 | Negative | -0.5074 |
|  | HLA-DPA1*02:01/DPB1*14:01 | 14 – 28 | GYFRLGVAMESMVKY | NetMHCIIpan | 0.03 | 0.4143 | Negative | -0.3416 |
|  | HLA-DPA1*02:01/DPB1*14:01 | 12 – 26 | LKGYFRLGVAMESMV | NetMHCIIpan | 0.03 | 0.9526 | Negative | -0.3270 |
|  | HLA-DPA1*02:01/DPB1*14:01 | 15 – 29 | YFRLGVAMESMVKYD | NetMHCIIpan | 0.07 | 0.5341 | Negative | -0.2952 |
|  | HLA-DPA1*02:01/DPB1*14:01 | 11 – 25 | WLKGYFRLGVAMESM | NetMHCIIpan | 0.1 | 1.0019 | Negative | -0.3327 |
|  | HLA-DPA1*02:01/DPB1*14:01 | 10 – 24 | DWLKGYFRLGVAMES | NetMHCIIpan | 0.73 | 0.8179 | **Positive** | 0.0948 |
|  | HLA-DRB1*04:05 | 10 – 24 | DWLKGYFRLGVAMES | Consensus (smm/nn/sturniolo) | 0.86 | 0.8179 | **Positive** | 0.0948 |
|  | HLA-DRB1*04:05 | 13- 27 | KGYFRLGVAMESMVK | Consensus (smm/nn/sturniolo) | 0.86 | 0.5969 | Negative | -0.5076 |
|  | HLA-DRB1*04:05 | 12 – 26 | LKGYFRLGVAMESMV | Consensus (smm/nn/sturniolo) | 0.86 | 0.9526 | Negative | -0.3279 |
|  | HLA-DRB1*04:05 | 11 - 25 | WLKGYFRLGVAMESM | Consensus (smm/nn/sturniolo) | 0.86 | 1.0019 | Negative | -0.4636 |
