# Supplementary material for: Engineering and design of promising T-cell-based multi-epitope vaccine candidates against leishmaniasis
Source: Sci Rep. 2023 Nov 8;13:19421. doi: 10.1038/s41598-023-46408-1 (PMC10632461; doi:10.1038/s41598-023-46408-1)
Supplement: Supplementary file 3 — Supplementary Table S2. [file 41598_2023_46408_MOESM3_ESM.docx]

**Supplementary Table S2**

**Leish-*App* and Leish-*Rpf* as two promising T-cell-based Multi-Epitope Vaccine Candidates Against Leishmaniasis**

**Supplementary Table S2.** Cytotoxic T-lymphocyte specific epitope prediction for six selected *L. major* vaccine candidate antigens and subsequent screening regarding immunogenicity and IFN-γ induction

|  |  |  |  |  |  |  |  |  |
| --- | --- | --- | --- | --- | --- | --- | --- | --- |
| Protein name | **MHC-I allele** | **Start** | **End** | **Length** | **Peptide** | **Percentile rank** | **Immunogenicity** | **IFN**-γ **production** |
| Histone H2A | HLA-B*07:02 | 9 | 17 | 9 | KPRHLLLAI | 0.02 | 0.04161 | Negative |
|  | HLA-A*68:01 | 25 | 33 | 9 | QVVKATISR | 0.02 | -0.1133 | Negative |
|  | HLA-A*02:01 | 47 | 55 | 9 | YLTTEVIEL | 0.02 | 0.35884 | Negative |
|  | HLA-A*03:01 | 22 | 30 | 9 | RLKDGLYRK | 0.01 | 0.002 | Negative |
|  | HLA-A*02:06 | 47 | 55 | 9 | YLTTEVIEL | 0.04 | 0.35884 | Negative |
|  | HLA-B*08:01 | 7 | 15 | 9 | RIKPRHLLL | 0.02 | -0.01615 | Negative |
|  | HLA-A*02:01 | 43 | 52 | 10 | ALLEYLTTEV | 0.04 | 0.21055 | Negative |
|  | HLA-A*68:01 | 24 | 33 | 10 | NQVVKATISR | 0.11 | -0.06476 | Negative |
|  | HLA-B*58:01 | 6 | 14 | 9 | RAARAELNF | 0.09 | 0.18399 | **Positive** |
|  | HLA-A*31:01 | 47 | 55 | 9 | KSKKKSAKR | 0.04 | -0.74571 | Negative |
| Cathepsin L | HLA-B*44:03 | 30 | 38 | 9 | SEAEFAARY | 0.01 | 0.32754 | **Positive** |
|  | HLA-B*44:02 | 30 | 38 | 9 | SEAEFAARY | 0.01 | 0.32754 | **Positive** |
|  | HLA-A*24:02 | 37 | 45 | 9 | RYLNGAAYF | 0.01 | 0.09058 | Negative |
|  | HLA-A*23:01 | 37 | 45 | 9 | RYLNGAAYF | 0.01 | 0.09058 | Negative |
|  | HLA-B*58:01 | 6 | 14 | 9 | SSERVMTAW | 0.03 | 0.0151 | Negative |
|  | HLA-B*57:01 | 6 | 14 | 9 | SSERVMTAW | 0.06 | 0.0151 | Negative |
|  | HLA-A*02:01 | 30 | 38 | 9 | LLTGYPVSV | 0.03 | -0.02916 | Negative |
|  | HLA-A*01:01 | 24 | 33 | 10 | AVDASSFMSY | 0.02 | -0.41029 | Negative |
|  | HLA-B*58:01 | 4 | 12 | 9 | SAVPDAVDW | 0.05 | 0.10847 | **Positive** |
|  | HLA-B*57:01 | 34 | 43 | 10 | SAVGNIESQW | 0.09 | 0.05775 | **Positive** |
| Cathepsin B | HLA-B*40:01 | 2 | 10 | 9 | GEKELMIEL | 0.01 | 0.02547 | **Positive** |
|  | HLA-B*44:03 | 21 | 29 | 9 | TEAVPPRNF | 0.01 | 0.0729 | Negative |
|  | HLA-B*44:02 | 21 | 29 | 9 | TEAVPPRNF | 0.01 | 0.0729 | Negative |
|  | HLA-A*68:01 | 21 | 29 | 9 | TTVSGLYAK | 0.03 | -0.11077 | Negative |
|  | HLA-A*11:01 | 21 | 29 | 9 | TTVSGLYAK | 0.01 | -0.11077 | Negative |
|  | HLA-A*01:01 | 8 | 16 | 9 | NTDWGDKGY | 0.02 | 0.12177 | Negative |
|  | HLA-A*24:02 | 24 | 32 | 9 | KYPPCPSTI | 0.02 | -0.19464 | Negative |
|  | HLA-B*15:01 | 49 | 58 | 10 | LVKYKGSTSY | 0.01 | -0.47035 | Negative |
|  | HLA-B*35:01 | 25 | 33 | 9 | YPPCPSTIY | 0.03 | -0.11386 | Negative |
|  | HLA-B*44:02 | 20 | 29 | 10 | STEAVPPRNF | 0.02 | 0.13073 | Negative |
| Grp78 | HLA-A*11:01 | 31 | 39 | 9 | SVTNPIIQK | 0.01 | 0.16521 | Negative |
|  | HLA-B*15:01 | 38 | 46 | 9 | KMKEISETF | 0.01 | 0.1118 | Negative |
|  | HLA-A*68:01 | 13 | 21 | 9 | ETVGGVMTK | 0.01 | -0.00616 | Negative |
|  | HLA-A*68:01 | 30 | 38 | 9 | EVSAMVLQK | 0.02 | -0.22351 | Negative |
|  | HLA-A*03:01 | 31 | 39 | 9 | SVTNPIIQK | 0.01 | 0.16521 | Negative |
|  | HLA-B*35:01 | 27 | 35 | 9 | EPTAAAIAY | 0.02 | 0.26208 | **Positive** |
|  | HLA-B*40:01 | 22 | 31 | 10 | RERVEAKNSL | 0.02 | -0.11118 | Negative |
|  | HLA-B*35:01 | 44 | 52 | 9 | QPSVLIQVF | 0.02 | 0.02868 | Negative |
|  | HLA-B*57:01 | 44 | 52 | 9 | TTYSVAGVW | 0.05 | -0.03792 | Negative |
|  | HLA-A*02:03 | 21 | 29 | 9 | KLIERNTQI | 0.01 | 0.15334 | Negative |
| Gp46 | HLA-B*58:01 | 3 | 11 | 9 | LTGPLPEEW | 0.01 | 0.1216 | **Positive** |
|  | HLA-B*58:01 | 39 | 47 | 9 | LTGTLPPTW | 0.01 | 0.04214 | Negative |
|  | HLA-B*57:01 | 39 | 47 | 9 | LTGTLPPTW | 0.01 | 0.04214 | Negative |
|  | HLA-B*57:01 | 3 | 11 | 9 | LTGPLPEEW | 0.01 | 0.1216 | **Positive** |
|  | HLA-B*58:01 | 27 | 35 | 9 | LTGTLPPEW | 0.01 | 0.07796 | **Positive** |
|  | HLA-B*07:02 | 13 | 21 | 9 | RPRAALLAV | 0.01 | 0.09733 | **Positive** |
|  | HLA-B*57:01 | 27 | 35 | 9 | LTGTLPPEW | 0.01 | 0.07796 | **Positive** |
|  | HLA-B*58:01 | 15 | 23 | 9 | LTGTLPSSW | 0.01 | -0.20746 | Negative |
|  | HLA-B*58:01 | 51 | 59 | 9 | LTGTIPEAW | 0.01 | 0.27658 | Negative |
|  | HLA-B*57:01 | 15 | 23 | 9 | LTGTLPSSW | 0.02 | -0.20746 | Negative |
| STI-1 | HLA-B*58:01 | 34 | 42 | 9 | IAYEGMEKW | 0.01 | -0.07425 | Negative |
|  | HLA-B*57:01 | 34 | 42 | 9 | IAYEGMEKW | 0.01 | -0.07425 | Negative |
|  | HLA-A*68:02 | 47 | 55 | 9 | EVMDKLHAI | 0.01 | -0.20496 | Negative |
|  | HLA-B*35:01 | 20 | 28 | 9 | VAMESMVKY | 0.02 | -0.37381 | Negative |
|  | HLA-B*35:01 | 46 | 54 | 9 | EPVKEKAVY | 0.02 | -0.25196 | **Positive** |
|  | HLA-A*68:01 | 35 | 44 | 10 | EASGALYSNR | 0.04 | -0.13918 | Negative |
|  | HLA-A*02:03 | 51 | 59 | 9 | KLHAINTKV | 0.03 | 0.08014 | Negative |
|  | HLA-B*44:03 | 15 | 24 | 10 | EEAKQLGNSF | 0.04 | -0.39812 | Negative |
|  | HLA-B*44:02 | 15 | 24 | 10 | EEAKQLGNSF | 0.03 | -0.39812 | Negative |
|  | HLA-B*40:01 | 34 | 43 | 10 | AEFYTRAIEL | 0.07 | 0.33013 | Negative |
